# Calcineurin depletion coincides with phosphorylated TDP-43 deposition in a mouse model of ALS/FTLD-TDP

**DOI:** 10.1186/s40478-025-02192-9

**Published:** 2026-01-03

**Authors:** Sarah M. Waldherr, Randall J. Eck, Joshua C. Hincks, Heather N. Currey, Matvey Goldberg, Pamela J. McMillan, Aleen D. Saxton, Heino J. Hulsey-Vincent, Caitlin S. Latimer, Brian C. Kraemer, Nicole F. Liachko

**Affiliations:** 1 Geriatrics Research Education and Clinical Center, Veterans Affairs Puget Sound Health Care System, S182, 1660 South Columbian Way, Seattle, WA 98108 USA; 2 Division of Gerontology and Geriatric Medicine, Department of Medicine, University of Washington, Seattle, WA 98104 USA; 3 Graduate Program in Neuroscience, University of Washington, Seattle, WA 98195 USA; 4 Department of Psychiatry and Behavioral Sciences, University of Washington, Seattle, WA 98195 USA; 5 Department of Laboratory Medicine and Pathology, University of Washington, Seattle, WA 98195 USA; 6 Department of Genome Sciences, University of Washington, Seattle, WA 98104 USA

**Keywords:** Calcineurin, CDC7, TDP-43, Amyotrophic lateral sclerosis (ALS), Frontotemporal lobar degeneration (FTLD), Neurodegeneration, Kinase, Phosphatase

## Abstract

Amyotrophic lateral sclerosis (ALS) and frontotemporal lobar degeneration (FTLD-TDP) exhibit predominantly cytoplasmic phosphorylated inclusions of the protein TDP-43 as the major neuropathological lesion. Phosphorylated TDP-43 can modify protein aggregation and promote neuronal dysfunction and neurodegeneration in models of ALS and FTLD-TDP. The phosphatase calcineurin has previously been shown to directly dephosphorylate TDP-43 *in vitro* and prevent accumulation of phosphorylated TDP-43 *in vivo* in *C. elegans*. However, it is unknown whether dysregulation of calcineurin contributes to increased TDP-43 phosphorylation and neurodegeneration in the mammalian brain. Here we show in an inducible mouse model of ALS/FTLD-TDP driven by expression and cytoplasmic mislocalization of human TDP-43 (rNLS8 mice), calcineurin protein decreases dramatically in the brain. This depletion coincides with increased levels of the TDP-43 kinase CDC7 and accumulation of phosphorylated TDP-43, and precedes frank neurodegeneration. Using brain-wide single nucleus RNA sequencing (snRNAseq) in symptomatic rNLS8 mice, we find cell-type selective reduced expression of catalytic and regulatory subunits of calcineurin predominantly in GABAergic and glutamatergic neurons. In mouse primary neuron culture and *C. elegans* models of ALS/FTLD-TDP, we demonstrate activation or overexpression of calcineurin protects against accumulation of phosphorylated TDP-43, neurotoxicity, and neurodegeneration. Taken together, our data suggests calcineurin dysregulation may be a major contributor to loss of brain resilience mechanisms against phosphorylated TDP-43. Restoring calcineurin activity may present a new target for intervening in TDP-43 proteinopathies, including ALS and FTLD-TDP.

**Supplementary Information:**

The online version contains supplementary material available at 10.1186/s40478-025-02192-9.

## Background

Amyotrophic lateral sclerosis (ALS) and frontotemporal lobar degeneration with transactive response DNA binding protein of 43 kDa (TDP-43) positive inclusions (FTLD-TDP) represent a clinical and pathological spectrum of neurodegenerative disease featuring phosphorylated aggregates of the protein TDP-43 [1]. These TDP-43 positive inclusions accumulate in neurons in the central nervous system, particularly in the motor cortex and spinal cord in ALS and in the frontal and temporal lobes in FTLD-TDP [2–5]. Mutations in the gene coding for TDP-43, *TARDBP*, cause some familial inherited cases of ALS, indicating dysfunction of TDP-43 leads to neurodegeneration [6]. TDP-43 pathology has recently been described in limbic-predominant age-related TDP-43 encephalopathy (LATE) and is present as a secondary pathology in a significant subset of patients with other neurodegenerative diseases, including Alzheimer’s disease (AD), chronic traumatic encephalopathy (CTE), Lewy body dementia (LBD), and Huntington’s disease (HD) [7]. The presence of co-morbid TDP-43 inclusions in these diseases likely contributes to worsened clinical and neuropathological outcomes. Therefore, understanding mechanisms contributing to pathological TDP-43 may be broadly relevant for treating both primary and secondary TDP-43 protein misfolding diseases (proteinopathies).

Disease-associated TDP-43 exhibits a number of post-translational modifications at sites not observed in healthy neurons. These include phosphorylation, acetylation, ubiquitination, SUMO-ylation, hypusination, and truncated protein lacking the normal N-terminus [3, 4, 8, 9]. Of these modifications, phosphorylation of TDP-43 at serines S409 and S410 (S409/S410) is the most well-recognized disease-associated epitope and is used diagnostically to identify TDP-43 proteinopathies. At least twenty disease-linked missense mutations in *TARDBP* have been identified that introduce or delete potential phosphorylation sites [10, 11]. Evidence from model systems has shown TDP-43 phosphorylation has a number of modifying effects on protein function, including altering TDP-43 subcellular localization, messenger RNA (mRNA) splicing activity, solubility, and aggregation [12–22]. In cell culture, *Caenorhabditis elegans* (*C. elegans*), and *Drosophila melanogaster* models of ALS, phosphorylation of TDP-43 promotes cytotoxicity and neurodegeneration [17, 23].

Both kinases and phosphatases have been implicated in the regulation of TDP-43 phosphorylation. The kinases casein kinase 1 (CK1), cell division cycle 7-related protein kinase (CDC7), mitogen-activated protein kinase 14 (p38α), inhibitory kappa B kinase beta (IKKβ), tau tubulin kinase 1 (TTBK1), and tau tubulin kinase 2 (TTBK2) directly phosphorylate TDP-43 *in vivo*, while the phosphatases protein phosphatase 1 (PP1) and calcineurin (CaN) directly dephosphorylate TDP-43 [12, 14, 24–28]. In particular, the phosphatase calcineurin has been shown to bind and dephosphorylate TDP-43 at pathological sites S409/S410 *in vitro* using purified human phosphorylated TDP-43 [20]. Calcineurin and TDP-43 physically interact in human brain lysate and co-localize in TDP-43 positive inclusions in ALS spinal cord motor neurons, suggesting calcineurin is recruited to dephosphorylate TDP-43 in human disease [20, 29]. Calcineurin activity is decreased in both sporadic and familial ALS patients, making calcineurin dysregulation a possible contributor to disease [30, 31]. In mammalian cell culture, pharmacological inhibition of calcineurin using the drugs FK506 or Cyclosporin A drives accumulation of phosphorylated TDP-43, and in *C. elegans* models of ALS, genetic loss of the sole calcineurin A homolog, *tax-6*, dramatically worsens motor phenotypes, phosphorylated TDP-43 accumulation, and neurodegeneration [20]. Although calcineurin can directly dephosphorylate TDP-43, it is unknown whether changes in calcineurin may contribute to accumulation of phosphorylated TDP-43 during ALS or FTLD-TDP disease course. Calcineurin functions as a heterodimer with a catalytic and a regulatory subunit. In humans and mice, there are three isozymes of the catalytic subunit (PPP3CA, PPP3CB, and PPP3CC) and two isozymes of the regulatory subunit (PPP3R1 and PPP3R2). The isozymes within each subunit are nearly identical at the amino acid level and function similarly, although there are cell-type differences in expression among them [32, 33]. In the brain, PPP3CA and PPP3R1 are predominant isozymes.

In this study, we find dramatic reduction in calcineurin from brain regions vulnerable to TDP-43 proteinopathy in an inducible mouse model of ALS and FTLD-TDP (rNLS8). Calcineurin depletion precedes or accompanies appearance of phosphorylated TDP-43 and significant neuron loss, becoming more severe as disease progresses to end stage. We identify cell-type specific expression changes of both catalytic and regulatory subunits of calcineurin in the brain. Finally, we demonstrate chemical or genetic activation of calcineurin protects against TDP-43 neurotoxicity in mouse primary neurons and *C. elegans* models of ALS/FTLD-TDP*.*

## Materials and methods

### Mouse strains

For these studies, we used the rNLS8 mouse model of ALS/FTLD-TDP [34]. This mouse strain employs two transgenes: a *NEFH*-tTA driver and a *tetO-*hTDP-43-ΔNLS responder, allowing inducible expression of constitutively cytoplasmic human TDP-43. The driver transgene was obtained from The Jackson Laboratory (stock # 025397) carrying the human neurofilament heavy polypeptide (NEFH) promoter transgene B6.Cg-Tg(*NEFH*-tTA)8Vle/J (*NEFH*-tTA), which is inserted into chromosome 12: 6,917,892–6,917,912 without any disruption of gene regions. The responder transgene was a generous gift of Dr. Virginia Lee, also available from Jackson Laboratory (stock # 014650), carrying the human TDP-43 complementary DNA (cDNA) with point mutations inactivating the nuclear localization signal (NLS) (*tetO-*hTDP-43-ΔNLS). Animals carrying both *tetO-*hTDP-43-ΔNLS and *NEFH*-tTA transgenes are referred to as rNLS8 mice. Their non-transgenic littermates and single transgenic littermates carrying either the *tetO-*hTDP-43-ΔNLS or the *NEFH*-tTA transgene were used as controls. Data from *NEFH*-tTA, *tetO-*hTDP-43-ΔNLS, and non-transgenic animals were pooled to form the control group as there were no statistical differences between the genotypes on any of the measures. Both male and female animals were included in rNLS8 and control groups and balanced for sex as much as possible. To induce expression of the rNLS8 transgene, animals were removed from doxycycline (DOX) by switching from chow containing 625 ppm DOX (LabDiet or BioServ) to regular chow (5058/PicoLab Mouse Diet 20). Animals ranged from 3 to 7 months old at removal from DOX chow. Brain tissue was harvested 1, 4, or 7 weeks after removal from DOX.

### Mouse brain tissue collection

For fixed tissue harvest, animals were anesthetized with pentobarbitol and transcardial perfusion was performed first with saline to remove blood from the brain then with 4% paraformaldehyde to fix the tissue. Brains were removed from the skull and stored in 80% ethanol. Fixed brains were embedded in paraffin, sectioned into coronal sections 9 microns thick, and stored at 4 °C until use. For fresh tissue, animals were euthanized by CO_2_ asphyxiation, and brains were either split down the midline into halves or dissected into regions. Tissue was snap frozen in liquid nitrogen and stored at -80 °C until use.

### Immunohistochemistry analysis

Animal brain sections were deparaffinized, rehydrated through a gradient of alcohol concentrations, and processed through antigen retrieval steps consisting of heat pretreatment in citrate buffer by either microwave or autoclave per antibody-specific protocols (calcineurin: microwave, neuronal marker Rbfox3 (NeuN): microwave, phosphorylated TDP-43 (pTDP-43): autoclave). Sections were treated for endogenous peroxidases with 3% hydrogen peroxide in PBS (pH 7.4) for 30 min at room temperature, blocked in 5% non-fat milk in PBS for one hour at room temperature, and incubated with one of the following primary antibodies overnight at 4 °C: calcineurin (CN-A1 mAb, Rockland 200-301-P23, 1:500), NeuN (A60 mAb, MilliporeSigma MAB377, 1:2000) and pTDP-43 (S409/410, clone 1D3, gift from Dr. Manuela Neumann, 1:400) [30]. The next day, biotinylated secondary goat anti-mouse or goat anti-rat antibody (Vector Laboratories, Inc., Newark, CA, USA) was applied for 45 min at room temperature. Finally, sections were incubated in an avidin–biotin complex with streptavidin-HRP (VECTASTAIN Elite ABC-HRP kit, Vector Laboratories, Inc., Newark, CA, USA) for one hour at room temperature and the reaction product was visualized with a 10 min treatment of 0.05% diaminobenzidine (DAB)/0.01% hydrogen peroxide in PBS. Negative controls consisted of full protocol except primary antibody. For additional rigor and to minimize batch effect artifacts, all DAB immunostaining within each timepoint (1, 4, and 7 weeks) and brain region (hippocampus, cortex, and striatum) were performed simultaneously, and statistical comparisons were made only within each DAB immunostaining assay. Digital images were obtained using a Leica DM6 microscope with a DFC 7000 digital camera and LAS X imaging software (Leica Microsystems, Wetzlar, Germany) and imported into Adobe Photoshop (Adobe Inc., San Jose, CA, USA). HALO digital image software (Indica Labs, Albuquerque, New Mexico, USA) was used to quantify immunoreactivity (IR). For quantification, all images were taken using the same exposure (8 ms). Brain sections were manually annotated around the regions of interest, average staining intensity for each antibody was determined to allow quantification without contribution of background staining, and a common threshold was then applied to all sections for that assay. Data represent the area of positive IR within the region of interest divided by the total annotated area. This value was then multiplied by the average optical density (OD) of IR to yield the final normalized IR area x OD. Representative images were chosen based on animals with quantification similar to the group average.

To quantify the number of hippocampal NeuN positive cells, slides were scanned on an Aperio AT2 digital pathology scanner (Leica Biosystems, Nussloch, Germany) at 20 × magnification. Using HALO digital image software v.3.6 (Indica Labs, Albuquerque, New Mexico, USA), the hippocampal CA3 region was manually annotated for each animal. A membrane segmentation model was trained to detect NeuN positive cells using HALO AI. The training set was generated from 22 animals and included 562 manually annotated NeuN positive cells and 67 regions of background staining. Once trained, the model was applied to experimental groups, followed by a blinded quality check of each slide. Cell density was calculated by dividing the number of detected cells by the annotated hippocampal CA3 area for each animal.

Cortical thickness of the grey matter was measured by annotating a perpendicular line from the outer pial surface of the cortex to the dorsal corpus callosum white matter tract at the approximate level of the motor cortex and cingulate cortex (approximately Bregma − 0.71 mm). Cortical area of the grey matter was measured by annotating the area from the outer pial surface of the cortex to the dorsal corpus callosum white matter tract at approximately Bregma -0.71 mm).

All immunohistochemistry (IHC) analyses were performed blinded to groups.

### Immunoblotting analysis

Fresh frozen mouse brain samples were thawed on ice, and suspended in 480 µL cold LS buffer (10 mM Tris, 5 mM EDTA, 10% sucrose, 1 mM DTT, 0.5 mM PMSF, and protease inhibitor cocktail (Roche cOmplete Mini)). Samples were then physically homogenized with disposable pestles in microcentrifuge tubes, and then sonicated on ice for 10 s three times at 60% microtip power to further disrupt tissue integrity and release proteins into the buffer. Sonicated samples were boiled for 5 min to denature proteins, and centrifuged at 16,100 g for 1–2 min to pellet debris. Lysate preparation was loaded onto 4 to 15% gradient pre-cast criterion SDS-PAGE gradient gels and transferred to PVDF membrane as recommended by the manufacturer (Bio-Rad Laboratories, Hercules, CA, USA). PVDF membranes were blocked in 5% milk in PBS before overnight incubation with primary antibody at 4 °C. The next day, PVDF membranes were washed in PBS with 0.1% Tween, incubated at room temperature with HRP-coupled secondary antibody for two hours, and washed in PBS with 0.1% Tween before visualization. Enhanced chemiluminescence substrate (Bio-Rad Laboratories, Hercules, CA, USA) was added to the PVDF membrane, and chemiluminescence signals were detected on film. Antibodies used include total TDP-43 (Abcam ab57105), phosphorylated TDP-43 (CosmoBio TIP-PTD-M01), calcineurin (Millipore MP 07-067), CDC7 (Abcam ab108332), and actin (Sigma Aldrich A4700). Secondary goat anti-mouse or goat anti-rabbit IgG (Jackson ImmunoResearch, West Grove, PA, USA) were the secondary antibody reagents used at a dilution of 1:1000. Relative intensity of chemiluminescence signals was measured from scanned film using ImageJ software.

### Single nucleus RNA sequencing (snRNAseq)

snRNAseq was performed on fresh frozen brains split down the midline into halves from five control and five rNLS8 mice at 4 weeks post-transgene induction. Female mice were used to minimize sex-based differences on gene expression. Nuclei isolation and fixation was performed as a fee-for-service by The Brotman Baty Institute for Precision Medicine at University of Washington (Seattle, WA, USA). Single-nucleus combinatorial indexing RNA-seq (sci-RNA-seq3) was performed as described [35]. In brief, three rounds of barcoding were used to tag transcripts with unique combinatorial indices traced back to individual nuclei. Sequencing was performed on an Illumina NovaSeq S4 flow cell at the University of Washington Northwest Genomics Center core sequencing facility (Seattle, WA, USA). Read alignment and gene count matrix generation was performed using an established pipeline (https://github.com/bbi-lab/bbi-dmux; https://github.com/bbi-lab/bbi-sci). The mouse GRCm39 build and annotation from Ensembl (v107) was used as the reference genome [36]. A count matrix data cell data set (cds) included data from all 10 mouse brains. The cds was processed to remove nuclei with unique molecular identifier counts greater than 15,000 or less than 200, or mitochondrial gene reads > 5%. Data were normalized using principal components analysis (PCA), dimensionality reduction using uniform manifold approximation and projection (UMAP), and grouping into clusters using community detection [37, 38]. Cds processing and analysis was performed using the Monocle3 package in R (version 1.4.26) and DESeq2 (version 1.48.1) for psuedobulk analysis, and Libra for pseudobulk matrix generation (version 1.0.0) in R (version 4.5.1) [39–41]. The Allen Institute for Brain Science tool MapMyCells was used to assign cell taxonomies clusters and subclusters using correlational mapping to the whole mouse brain (CCN20230722) [42]. Gene ontology (GO) was performed using the Database for Annotation, Visualization, and Integrated Discovery (DAVID) (version v2025_1) [43, 44].

### Quantitative reverse transcription PCR (qRT-PCR)

Mouse brains were harvested, dissected into regions, snap frozen in liquid nitrogen, and stored at − 80 °C until use. Total RNA was extracted from samples using TRI Reagent (Thermo Fisher Scientific, Waltham, MA, USA) according to manufacturer’s recommendations. Single-stranded cDNA was synthesized from 1 μg of RNA using the commercial kit iScript gDNA Clear cDNA Synthesis Kit (Bio-Rad Laboratories, Hercules, CA, USA). qRT-PCR assays were performed using the iTaq Universal SYBR Green Supermix kit (Bio-Rad Laboratories, Hercules, CA, USA) on a CFX Connect Real-Time PCR Detection system (Bio-Rad Laboratories, Hercules, CA, USA).

### Mouse primary neurons

Primary cortical neurons were isolated from post-natal day 0/1 C57BL/6 pups based on the protocol described in [45] with some modifications. In brief, removed brains were incubated on ice in Neurobasal A (NB-A) and supplemented with 5 mM HEPES and 30 mM glucose. Brains were dissected under a microscope to isolate the cortex and triturated in HBSS(-) with 5 mM HEPES, 30 mM glucose, 2.5% trypsin, and 0.01% DNAse I. Cells were washed and plated in poly-D-lysine coated cell culture dishes in NB-A with B27 supplementation. For each experiment the integrity of neuronal cell bodies and processes were visualized using DIC enabled white light microscopy prior to initiating experiments and prior to cell harvest for immunoblotting. At one week *in vitro*, cells were pre-treated with 250 µM nickel chloride (NiCl_2_) for two hours, followed by treatment with 75 µM ethacrynic acid (EA) to induce phosphorylated TDP-43. After three hours, cells were harvested, washed, snap frozen in liquid nitrogen, and stored at − 80 °C until use.

### *C. elegans* strains

*C. elegans* strains were maintained at 20 °C on Nematode Growth Media (NGM) agar prepared according to standard protocols [46] and seeded with OP50 *E. coli.* N2 are wildtype non-transgenic (non-Tg) controls. KJ306 *tax-6(jh107)* were crossed with CK426 *bkIs426[Psnb-1::TDP-43(A315T)*+*Pmyo-2::mCherry]* (referred to as TDP-43 Tg) to generate CK861 *tax-6(jh107); bkIs426[Psnb-1::TDP-43(A315T)*+*Pmyo-2::mCherry]* [referred to as TDP-43 Tg; *tax-6(jh107)*]. Transgenic WT *tax-6* overexpression (o/ex) lines were generated by microinjection of 50 µg/mL *Ptax-6*::TAX-6 and 20 µg/mL *Pelt-2*::mCherry co-injection marker. Extrachromosomal arrays were integrated with gamma irradiation (dose equivalent to ~ 3600 rad over 10 min). Integrated strains were backcrossed two times to N2 to generate strain CK1065 *bkIs1064[Ptax-6::TAX-6*+*Pelt-2::mCherry]* (referred to as *tax-6 *o/ex), and crossed with CK426 *bkIs426[Psnb-1::TDP-43(A315T)*+*Pmyo-2::mCherry]* and CK423 *bkIs423[Psnb-1::TDP-43(M337V)*+*Pmyo-2::mCherry]* to generate CK1125 *bkIs1125[Ptax-6::TAX-6*+*Pelt-2::mCherry*+*Psnb-1::TDP-43(A315T)*+*Pmyo-2::mCherry]* [referred to as TDP-43 Tg; *tax-6*(o/ex)] and CK1126 *bkIs1125[Ptax-6::TAX-6*+*Pelt-2::mCherry*+*Psnb-1::TDP-43(M337V)*+*Pmyo-2::mCherry]* (referred to as TDP-43 Tg; *tax-6 *o/ex 2).

### Radial locomotion behavioral analysis

*C. elegans* locomotion was assessed using published methods [47]. *C. elegans* were developmentally synchronized using a timed egg-lay and grown until all reached L4 stage at 20 °C. Developmentally synchronized animals were placed at the center of a 100 mm NGM 5X PEP plate. Animals were allowed to move freely for one hour, and the radial distance traveled from the start point was recorded as radial dispersion in mm. Two biological replicates were included per experiment, and at least three experimental replicates with at least 30 animals per replicate were analyzed for statistical significance.

### Electropharyngeogram analysis

*C. elegans* pumping electrophysiology was evaluated using a ScreenChip System (InVivo Biosystems, Eugene, OR, USA). Day one adult *C. elegans* were pre-incubated in M9 buffer containing 10 mM 5HT (Sigma-Aldrich, Burlington, Massachusetts, USA) for 20 min to stimulate pharyngeal pumping [48]. *C. elegans* were then individually loaded into the microfluidic recording device, and pharyngeal muscle and neuron action potentials were recorded for two minutes using NemAquire software (InVivo Biosystems, Eugene, OR, USA). Action potential statistics, including frequency and duration, were computed using NemAnalysis software (InVivo Biosystems, Eugene, OR, USA). Three experimental replicates with at least 10 animals per replicate were analyzed for statistical significance.

### Neurodegeneration analysis

Analysis of *C. elegans* neurodegeneration was performed using a transgenic reporter strain, EG1285 *oxIs12[Punc-47::GFP*+*lin-15(*+*)]* [49], with fluorescently marked VD and DD type inhibitory motor neurons as previously described [50]. *C. elegans* were developmentally synchronized using a timed egg-lay and grown until all reached day one of adulthood. *C. elegans* were mounted on glass slides with a 2% agarose gel pad and 10 µL 0.1% sodium azide. Green fluorescent protein (GFP) marked gamma-aminobutyric acid (GABA)ergic neurons were visualized via fluorescence microscopy. The number of intact neurons were counted on a DeltaVision Elite (GE, Issaquah, WA, USA) imaging system using an Olympus 60 × oil objective. Neurodegeneration was plotted as the average number of neurons lost from the 19 possible neurons analyzed. Three experimental replicates with at least 10 animals per replicate were analyzed for statistical significance.

### Statistical analysis

Statistical significance for all assays was determined using GraphPad Prism version 10.4.1 statistical software (GraphPad Software, Inc., Boston, MA, USA). Statistical significance is demarcated in figures as ns: *p* > 0.05, *: *p* ≤ 0.05, **: *p* ≤ 0.01, ***: *p* ≤ 0.001, and ****: *p* ≤ 0.0001. The details about experimental design and statistics used in different data analyses performed in this study are given in the respective sections of methods and figure legends.

## Results

### Calcineurin levels are reduced throughout the brain in response to cytoplasmic TDP-43

**Fig. 1:**
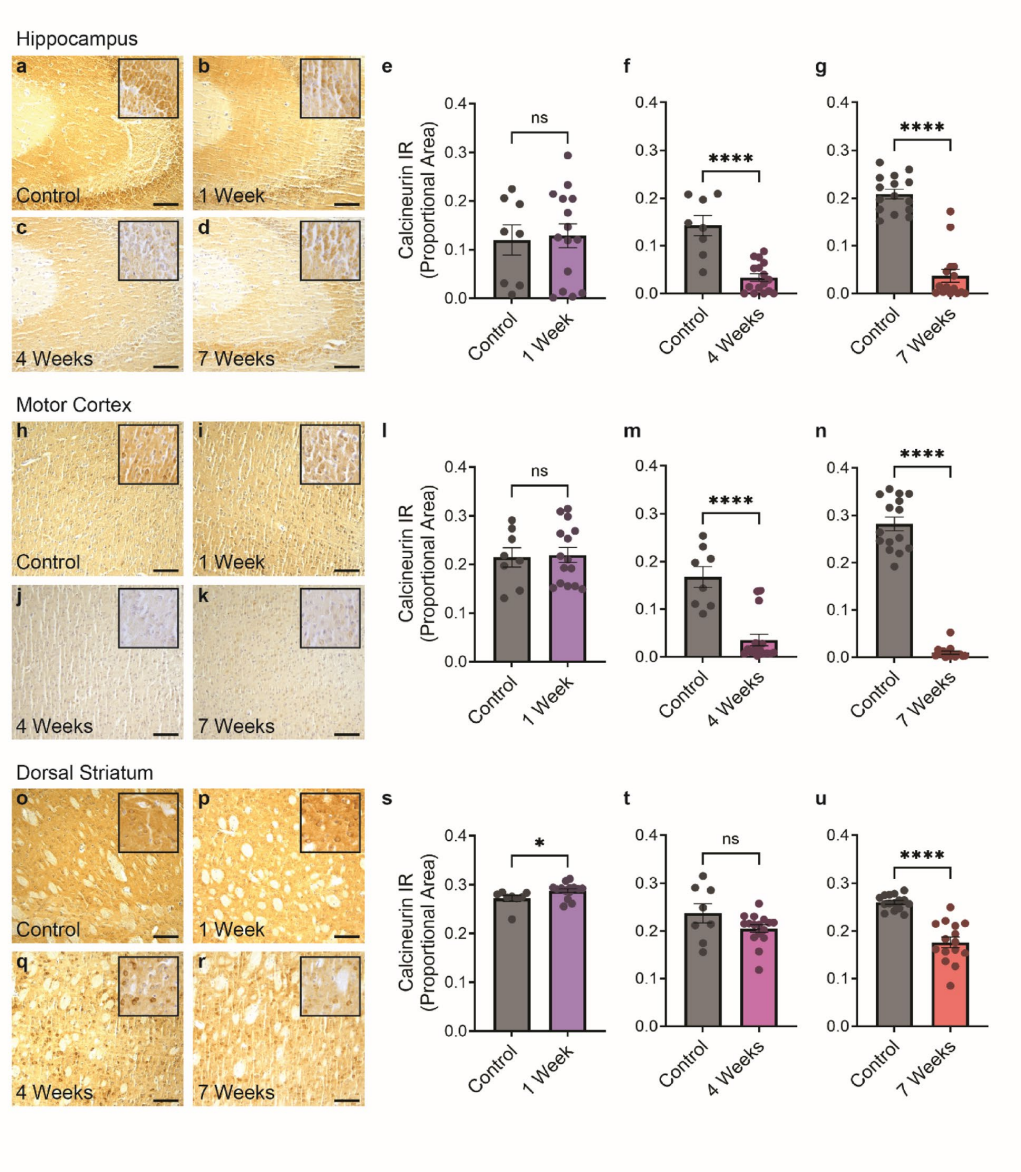
Reduced calcineurin in rNLS8 mouse brain at symptomatic timepoints. Representative images of calcineurin immunostaining in control animals, and rNLS8 animals at 1 week, 4 weeks, and 7 weeks post-induction. **a**–**d** hippocampus, **h**–**k** motor cortex, **o**–**r** dorsal striatum. Scale bars: 100 µm. Insets show higher magnification detail. Immunoreactivity quantification at 1 week, 4 weeks, and 7 weeks post-induction in hippocampus (**e**–**g**), motor cortex (**l**–**n**), and dorsal striatum (**s**–**u**). Statistical analysis is by unpaired *t*-test, two-tailed [*n* = 8 control, 15 rNLS8 animals (1 week); 8 control, 16 rNLS8 animals (4 weeks); 15 control, 15 rNLS8 animals (7 weeks); ns: *p* > 0.05, *: *p* ≤ 0.05, and ****: *p* ≤ 0.0001]. All bar graphs represent mean ± SEM

The phosphatase calcineurin has been shown to protect against phosphorylated TDP-43 accumulation *in vitro* and in *C. elegans* models, but dynamics of calcineurin have not been examined in vertebrate models of ALS/FTLD-TDP. To examine calcineurin expression in the brain in response to pathological TDP-43, we utilized an established mouse model that features neurofilament heavy chain (NEFH) promoter-driven inducible expression of mutant human TDP-43 with a deletion of its nuclear localization signal (hTDP-43ΔNLS), referred to as rNLS8 mice [34]. Following transgene induction, this mouse model accumulates cytoplasmic, hyperphosphorylated TDP-43 in the brain and spinal cord, progressive motor dysfunction, neurodegeneration, and shortened lifespan. The rapid tempo of this model results in neurological symptoms evident by 2 weeks post-transgene induction, motor phenotypes by 4 weeks post-induction, and neuron loss by 6 weeks post-induction. To assess brain-region specific dynamics of calcineurin protein levels during disease, we assembled cohorts of rNLS8 and control animals at three timepoints: 1 week post-induction (pre-symptomatic stage, prior to development of overt symptoms), 4 weeks post-induction (symptomatic stage, an intermediate point in disease progression), and 7 weeks post-induction (end-stage). We performed immunohistochemistry (IHC) on paraffin-embedded fixed tissue from the hippocampus, primary and secondary motor cortex, and dorsal striatum (caudate nucleus and putamen) from each of these post-induction timepoints. Consistent with previously published data, cytoplasmic phosphorylated TDP-43 inclusions are present at 4 weeks and 7 weeks post-induction (Supplementary Fig. 1). Calcineurin localization appears predominantly cytoplasmic in all regions and timepoints surveyed. However, we found significant overall depletion of calcineurin in the hippocampus and motor cortex by 4 weeks post-induction, with further depletion at 7 weeks post-induction in the regions analyzed (Fig. 1a–n). In the dorsal striatum, we found a delayed depletion of calcineurin that was significantly reduced by 7 weeks post-induction in the region analyzed (Fig. 1o–u), suggesting there are brain region-specific differences in tempo and degree of calcineurin depletion.

**Fig. 2:**
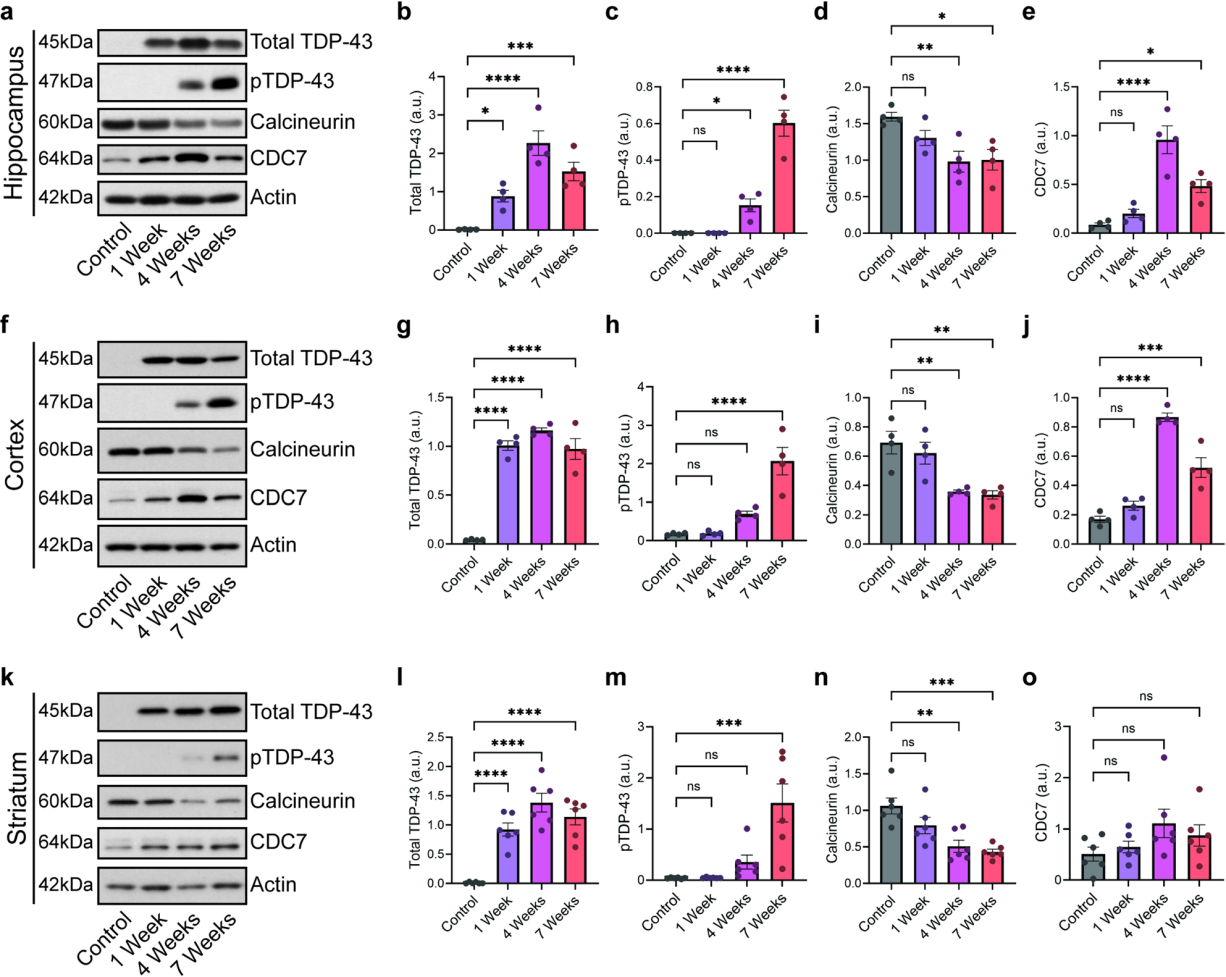
Calcineurin and CDC7 protein level changes correspond to onset of TDP-43 phosphorylation in rNLS8 mouse brain. Representative immunoblots and densitometry analysis of chemiluminescence signals for total TDP-43, phosphorylated TDP-43 (pTDP-43), calcineurin, CDC7, and actin for control animals, and rNLS8 animals at 1 week, 4 weeks, and 7 weeks post-induction. Quantification is normalized to actin. **a**–**e** hippocampus, **f**–**j** cortex, and **k**–**o** striatum. Statistical analysis is by one-way ANOVA, followed by Tukey’s post-test (*n* = 4–6 control animals and 4–6 rNLS8 animals for 1 week, 4 weeks, and 7 weeks post-transduction; ns: *p* > 0.05, *: *p* ≤ 0.05, **: *p* ≤ 0.01, ***: *p* ≤ 0.001, and ****: *p* ≤ 0.0001). All bar graphs represent mean ± SEM

To confirm these data, we performed semi-quantitative immunoblotting on the hippocampus, cortex, and striatum (Fig. 2). In all regions assessed, we found increases in total TDP-43 in rNLS8 animals beginning at 1 week post-induction (Fig. 2a, b, f, g, k, l). Total TDP-43 levels in rNLS8 animals were well above levels of endogenous mouse TDP-43, which was only detected in the control group lanes following overexposure of the immunoblots relative to the rNLS8 lanes (Supplementary Fig. 2). Phosphorylated TDP-43 was detected by 4 weeks post-induction in all three regions (Fig. 2a, c, f, h, k, m) and increased by 7 weeks post-induction. Calcineurin levels were modestly decreased in all three regions beginning at 4 weeks post-induction, and this decrease was sustained at 7 weeks post-induction (Fig. 2a, d, f, i, k, n). The timing of calcineurin depletion corresponded to the appearance of phosphorylated TDP-43 at 4 weeks post-induction, suggesting loss of calcineurin may permit phosphorylated TDP-43 accumulation.

TDP-43 phosphorylation is regulated by both kinases and phosphatases [12, 14, 20, 24, 25, 51]. Inhibition of the TDP-43 kinase CDC7 has previously been shown to protect against accumulation of TDP-43 phosphorylation and subsequent disease phenotypes in mouse and cell culture models [12, 52, 53]. To examine whether CDC7 levels change in response to cytoplasmic TDP-43 in rNLS8 mice, we probed immunoblots from 1, 4, and 7 weeks post-induction. We found CDC7 levels significantly increase in the hippocampus and cortex, but not the striatum, by 4 weeks post-induction (Fig. 2a, e, f, j, k, o), suggesting there may be region-specific responses to cytoplasmic TDP-43.

To determine whether depletion of calcineurin, increased CDC7, and accumulation of phosphorylated TDP-43 corresponds to onset or degree of neurodegeneration in rNLS8 mice, we performed several measures of brain integrity in vulnerable regions. In the hippocampus, we assessed neuronal density as detected by neuronal marker Rbfox3 (NeuN) immunostaining. We found significantly decreased number of neurons as measured by total NeuN immunoreactivity and NeuN positive cell counts in the CA3 region of the hippocampus at 7 weeks post-induction (Supplementary Fig. 3). In the cortex, we used NeuN immunoreactivity to detect grey matter. We measured motor and cingulate cortex thickness as well as overall cortical area, and found a significant reduction in rNLS8 compared to controls at both 4 and 7 weeks post-induction, indicating cortical atrophy (Supplementary Fig. 4), consistent with previously published analyses of neurodegenerative brain changes in rNLS8 mice [34]. These data support neurodegeneration onset coinciding with changes in TDP-43 phosphorylation.

**Fig. 3:**
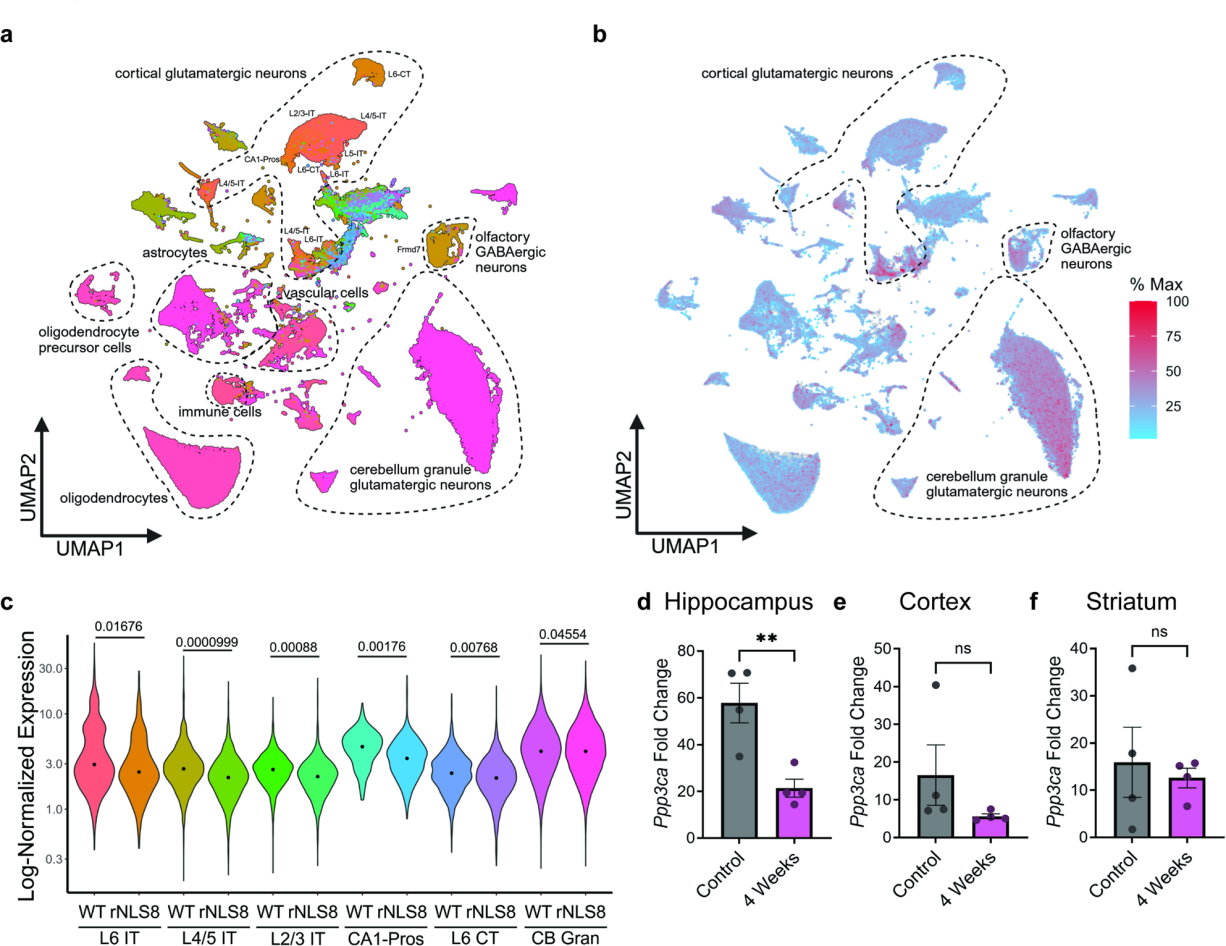
Reduced calcineurin transcription in symptomatic rNLS8 mouse brain. **a** UMAP showing cluster distribution of single nucleus RNA sequencing(snRNAseq) combined from 5 control animals and 5 rNLS8 animals at 4 weeks post-induction. **b** Average expression of calcineurin heterodimer catalytic and regulatory genes (Ppp3ca, Ppp3cb, Ppp3cc, Ppp3r1, Ppp3r2) throughout UMAP space. **c** Log-normalized expression of the calcineurin catalytic subunit Ppp3ca in control (WT) and rNLS8 animals in cortical layer 6 intratelencephalic-projecting glutamatergic neurons (L6 IT), cortical layer 4/5 intratelencephalic-projecting glutamatergic neurons (L4/5 IT), cortical layer 2/3 intratelencephalic-projecting glutamatergic neurons (L2/3 IT), hippocampal CA1 glutamatergic neurons (CA1-Pros), cortical layer 6 corticothalamicprojecting glutamatergic neurons (L6 CT), and cerebellar granule glutamatergic neurons (CB Gran). Significance values are p adj values generated from cluster level pseudobulk analysis with DESeq2. **d–f** qRT-PCR of the calcineurin catalytic subunit Ppp3ca mRNA levels in **d** hippocampus, **e** cortex, and **f** striatum. Statistical analysis is by unpaired t-test, two-tailed (n = 4 animals; N = 3 technical replicates; ns: p > 0.05, **: p ≤ 0.01). All bar graphs represent mean ± SEM

Protein level decreases of calcineurin and increases of CDC7 may be driven by transcriptional changes. To test this, we performed and analyzed single nucleus RNA sequencing (snRNAseq) data of nuclei isolated from throughout the brain of rNLS8 mice at 4 weeks post-induction. Following quality control processing to eliminate low quality nuclei, the dataset included 118,147 cells from 5 control mouse brains and 115,567 cells from 5 rNLS8 mouse brains. Calcineurin functions as a heterodimer, with three catalytic subunit isozymes (*Ppp3ca, Ppp3cb, Ppp3cc)* and two regulatory subunit isozymes (*Ppp3r1, Ppp3r2*). In this dataset, calcineurin complex subunits have strong expression in glutamatergic and gamma-aminobutyric acid (GABA)ergic neurons (Fig. 3a, b). When comparing calcineurin expression across 43 transcriptionally defined subclusters with > 500 cells per cluster via a pseudobulk differential gene expression analysis approach, *Ppp3ca* is the major subunit decreased in 8 neuronal clusters representing 105,241 cells, mapped as glutamatergic neurons in the cortex, hippocampus, and cerebellum, and GABAergic cells in the olfactory bulb (Fig. 3c, Table 1, and Supplementary Table 1). To confirm these data, we performed quantitative reverse transcription PCR (qRT-PCR) on bulk tissue from hippocampus, cortex, and striatum from 4 week post-induction animals. We found significant reduction in calcineurin in the hippocampus, but not the cortex or striatum (Fig. 3d-f). However, this may be due to confounding effects of bulk tissue in detecting differences present in a select subset of cells. We also surveyed other known TDP-43 kinases and phosphatases in our dataset, and found increased *Csnk1d* (CK1δ), *Ttbk1*, and *Pp1cb* (PP1ß), and decreased *Cdc7*, *Csnk1a1 (CK1α)*, and *Mapk11* in a small subset of clusters (Table 1, Supplementary Table 1).

**Table 1:** Differentially expressed TDP-43 kinases and phosphatases in symptomatic rNLS8

Gene name	Protein name	Subcluster/cell type	Ave Fold change	Ave p adj value	Cell count
Cdc7	Cell division cycle 7	Cerebellar granule glutamatergic neurons	− 0.498	2.58E−02	78,444
Csnk1a1	Casein kinase 1α	Hippocampus CA1 prosubiculum glutamatergic neurons	− 0.579	5.56E−05	847
Mapk11	Mitogen-activated protein kinase 11	Layer 6 corticothalamic-projecting glutamatergic cortical neuron	− 0.823	4.69E−04	4391
Mapk11	Mitogen-activated protein kinase 11	Layers 2/3, 4/5, 6 intratelencephalic-projecting glutamatergic cortical neurons	− 0.809	1.39E−02	14,580
Ppp3ca	Protein phosphatase 3 (calcineurin), catalytic subunit α	Hippocampus CA1 prosubiculum glutamatergic neurons	− 0.663	1.76E−03	847
Ppp3ca	Protein phosphatase 3 (calcineurin), catalytic subunit α	Olfactory blub, inner layers (mitral cell, internal plexiform and granule cell layers), FERM domain containing 7 GABAergic neuron	− 0.638	3.69E–03	6443
Ppp3ca	Protein phosphatase 3 (calcineurin), catalytic subunit α	Layers 2/3, 4/5, 5, 6 intratelencephalic-projecting glutamatergic cortical neuron	− 0.390	1.02E−02	15,116
Ppp3ca	Protein phosphatase 3 (calcineurin), catalytic subunit α	Layer 6 corticothalamic-projecting glutamatergic cortical neuron	− 0.393	7.68E−03	4391
Ppp3ca	Protein phosphatase 3 (calcineurin), catalytic subunit α	Cerebellar granule glutamatergic neurons	− 0.383	4.55E−02	78,444
Ppp3cb	Protein phosphatase 3 (calcineurin), catalytic subunit β	Hippocampus CA1-prosubiculum glutamatergic neurons	− 0.514	1.44E−02	847
Ppp3r1	Protein phosphatase 3 (calcineurin), regulatory subunit B,α	Hippocampus CA1-prosubiculum glutamatergic neurons	− 0.858	1.01E−02	847
Csnk1d	Casein kinase 1δ	Layer 4/5 intratelencephalic-projecting glutamatergic cortical neuron	0.346	2.95E−02	8018
Ppp1cb	Protein phosphatase 1 (PP1), catalytic subunit ß	Layers 2/3, 4/5 intratelencephalic-projecting glutamatergic cortical neurons	0.536	2.92E–03	5965
Ppp1cb	Protein phosphatase 1 (PP1), catalytic subunit ß	Layer 6 corticothalamic-projecting glutamatergic cortical neuron	0.537	1.30E−02	4391
Ppp1cb	Protein phosphatase 1 (PP1), catalytic subunit ß	Astrocyte-telencephalon	0.985	6.68E−03	7322
Ttbk1	Tau tubulin kinase 1	Layer 4/5 intratelencephalic-projecting glutamatergic cortical neuron	0.349	1.62E−02	8018
Ttbk2	Tau tubulin kinase 2	Layer 4/5 intratelencephalic-projecting glutamatergic cortical neuron	0.539	4.24E−02	8018

The snRNAseq dataset provides a wealth of information about cell-type specific gene expression changes in response to cytoplasmic TDP-43. Although not the main focus of this study, we found glutamatergic neuron populations in multiple cortical layers significantly differentially express hundreds of genes (|log| >|1.0| & *p adj* < 0.05), in line with human disease literature where these are known vulnerable neuronal populations (Supplementary Table 1) [54, 55]. As representative of these glutamatergic neurons, we highlight layer 4/5 intratelencephalically projecting neurons, which had the most gene expression differences in rNLS8 compared to controls, including significant decreases in calcineurin (*Ppp3ca*) (Table 1, Supplementary Tables 1, 2). In these cells, gene ontology (GO) highlights the disruption of several disease-relevant biological pathways such as the upregulation of genes involved in L-serine metabolism and the endoplasmic reticulum unfolded protein response (including the transcription factors *Atf3* and *Atf4*) as well as the downregulation of genes involved in synaptic transmission and axon guidance (including *Egr2* and *Sema3A*) (Supplementary Fig. 5, Supplementary Table 2).

A recent study identified global quantitative protein changes from the cortex of rNLS8 mice across symptomatic stages and during recovery [56]. Using their open-access webtool (TDP-map), we queried for significant protein changes in known TDP-43 kinases and phosphatases. Of the 25 proteins queried, 18 were detected in the dataset (Supplementary Table 3). Consistent with our snRNAseq data, the calcineurin isozymes Ppp3ca, Ppp3cb, and Ppp3r1 were all significantly decreased and the kinase Ttbk1 was significantly increased in rNLS8 mice at 4 weeks post-induction compared to controls. Additional changes identified were a significant decrease in CK1γ (Csnk1g3), p38 (Mapk14), and Ikk (Ikbkg). The proteomic dataset includes timepoints at 1, 2, 4, and 6 weeks post-induction, and at a recovery stage where the TDP-43 transgene was expressed for 6 weeks and then turned off for 2 weeks. Across disease timepoints, Ppp3ca, Ppp3cb, Csnk1g3, and Mapk14 were consistently downregulated and Ttbk1 was consistently upregulated. There were also transient decreases in the calcineurin isozyme Ppp3cc at 1 and 2 weeks post-induction and increases in CK1δ at 2 and 6 weeks post-induction. Taken together, this independent rNLS8 proteomic dataset confirms protein level changes in TDP-43 kinases and phosphatases with gene expression changes in our snRNAseq data.

### Calcineurin activation is protective against TDP-43 phosphorylation and neuronal dysfunction

**Fig. 4:**
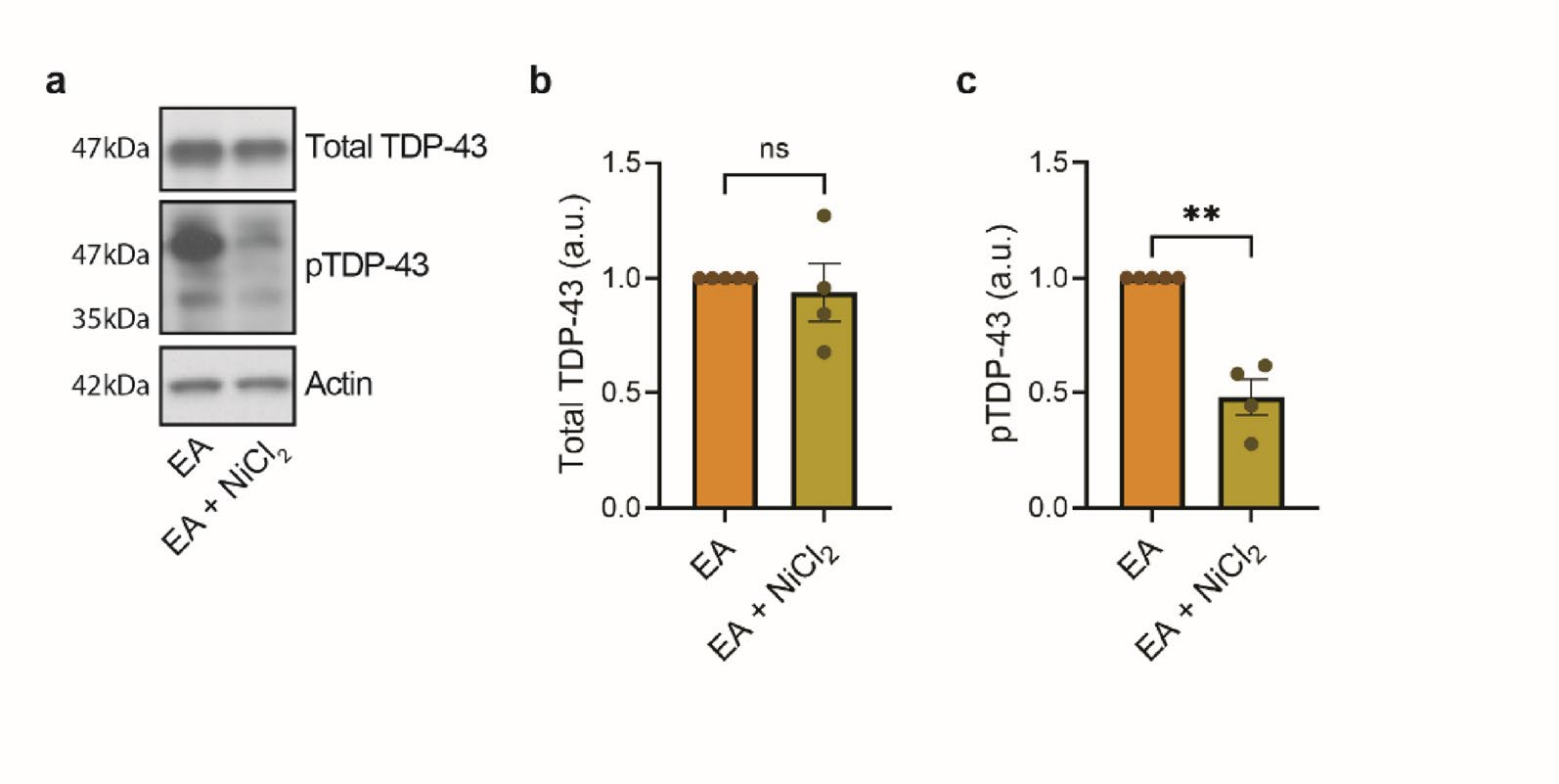
Activation of calcineurin protects against TDP-43 phosphorylation in mouse primary neurons. C57BL/6 mouse primary cortical neurons were cultured for 7 days and pretreated with either 0 µM or 250 µM nickel chloride (NiCl_2_) for 3 h to activate calcineurin. Cells were then exposed to 75 µM ethacrynic acid (EA) for 3 h to induce TDP-43 phosphorylation. **a** Representative immunoblots for total TDP-43, phosphorylated TDP-43 (pTDP-43), and actin. **b**–**c** Densitometry analysis of **b** total TDP-43 and **c** phosphorylated TDP-43 chemiluminescence signals normalized to actin and to control samples within each set. Statistical analysis is by paired *t*-test, two-tailed (*n* = 4 independent experiments; ns: *p* > 0.05 and **: *p* ≤ 0.01). All bar graphs represent mean ± SEM

Although we observe calcineurin depletion during disease progression in rNLS8 mice, artificially increasing calcineurin activity may protect against neurotoxic consequences of TDP-43 phosphorylation. In fact, we previously found increasing calcineurin activity via treatment with nickel chloride (NiCl_2_) in a HEK293 cell culture model protected against accumulation of phosphorylated TDP-43 [20]. However, it is unknown whether similar exogenous activation of calcineurin would protect against TDP-43 phosphorylation in neurons. C57BL/6 primary neurons do not accumulate significant levels of phosphorylated TDP-43 in the absence of a phosphorylated TDP-43 triggering condition as detected by immunoblot (Supplementary Fig. 6a–d). To stimulate accumulation of TDP-43 phosphorylation, we utilized a chemical trigger, ethacrynic acid (EA), which has previously been shown to drive TDP-43 phosphorylation [20, 57]. We treated cultured C57BL/6 mouse primary neurons with calcineurin-activating NiCl_2_ [58, 59], followed by treatment with EA to induce TDP-43 phosphorylation. We found a significant decrease in accumulation of phosphorylated TDP-43 in neurons that had exogenously activated calcineurin (Fig. 4, Supplementary Fig. 7e−h).

**Fig. 5:**
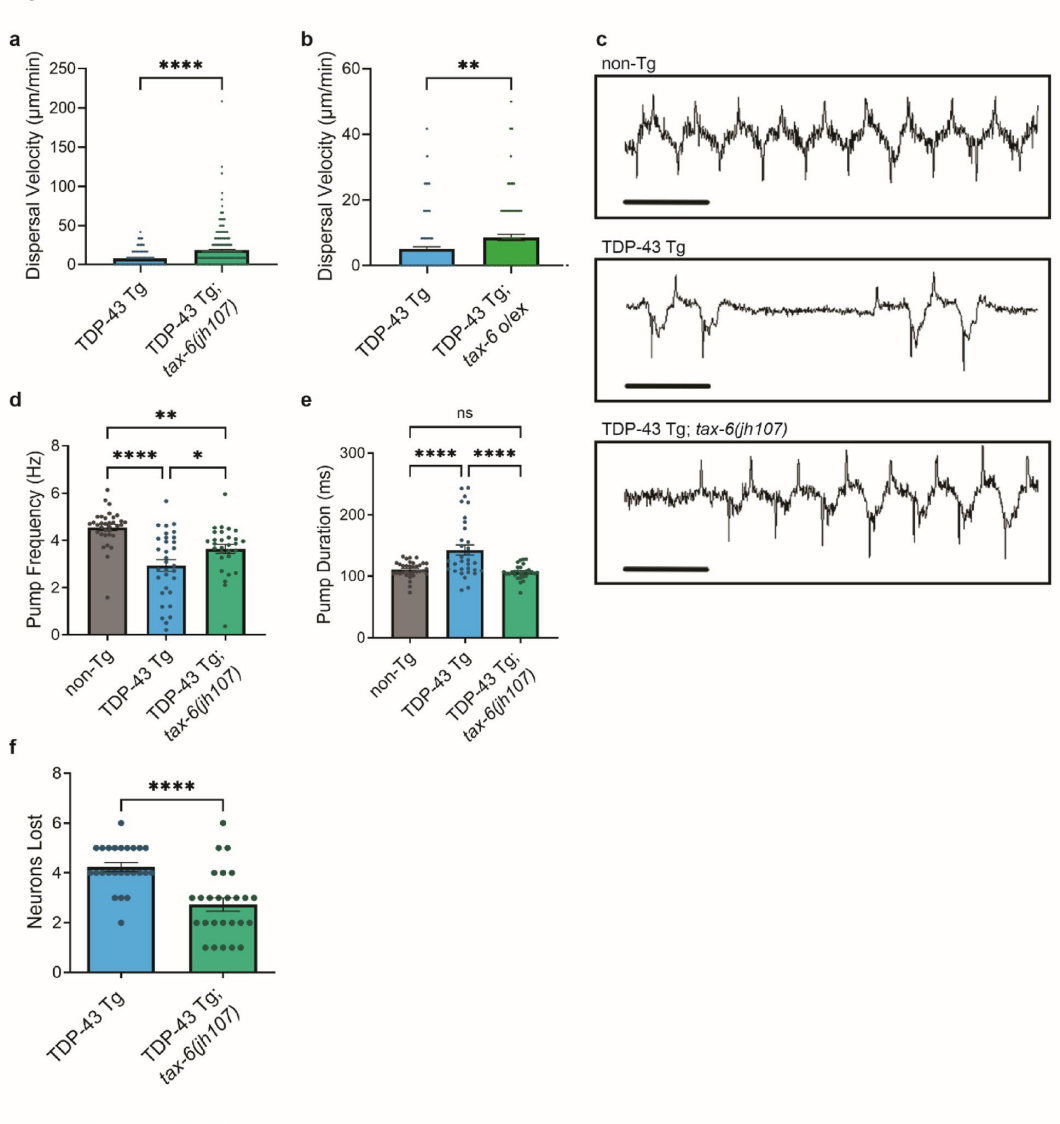
Activation of calcineurin protects against TDP-43 neurotoxicity in *C. elegans*. **a** A gain-of-function mutation in the *C. elegans* calcineurin homolog [*tax-6(jh107)*] improves TDP-43 transgenic *C. elegans* (TDP-43 Tg) motor dysfunction. Dispersal velocity of developmentally staged L4 larvae was measured by calculating the linear distance traveled from a designated central starting point over time. Statistical analysis is by unpaired *t*-test, two-tailed (*n* > 100 animals; *N* = 3 independent experiments; ****: *p* ≤ 0.0001). **b** A transgene overexpressing wildtype TAX-6 (*tax-6 o/ex*) improves TDP-43 Tg motor dysfunction, as measured by dispersal velocity. Statistical analysis is by unpaired *t*-test, two-tailed (*n* > 90 animals; N = 3 independent experiments, **: *p* ≤ 0.01). **c** Representative electropharyngeogram (EPG) recording traces from non-Tg, TDP-43 Tg, and TDP-43 Tg; *tax-6(jh107)* animals. Traces are 2 s; bar is 0.4 s. Frequency (**d**) and duration (**e**) of *C. elegans* pharyngeal pumping was evaluated using EPG recordings of neuron and muscle electrical signaling. *tax-6(jh107)* partially restores pharyngeal pumping frequency and restores pharyngeal pumping duration in TDP-43 Tg animals. Statistical analysis is by one-way ANOVA, followed by Tukey’s post-test (*n* ≥ 29 animals; *N* = 3 independent experiments; ns: *p* > 0.05, *: *p* ≤ 0.05, **: *p* ≤ 0.01, ****: *p* ≤ 0.0001). **f** GFP-labeled D-type GABAergic motor neurons were counted during late day 1 of adulthood *in vivo* in living animals. TDP-43 Tg animals had an average of 14.6 out of 19 total neurons (13 VD + 6 DD type neurons). TDP-43 Tg; *tax-6(jh107)* gain-of-function animals averaged 16.1 neurons per animal. Statistical analysis is by unpaired *t*-test, two-tailed (*n* ≥ 25 animals; ****: *p* ≤ 0.0001). All bar graphs represent mean ± SEM

To test whether increased calcineurin activity protects against TDP-43 neurotoxicity *in vivo*, we utilized a *C. elegans* model of familial ALS that pan-neuronally expresses human TDP-43 with the A315T mutation (referred to as TDP-43 Tg). TDP-43 Tg animals exhibit progressive motor dysfunction (uncoordination) and GABAergic motor neuron degeneration [17]. *C. elegans* have a single calcineurin catalytic subunit homolog called *tax-6*, and *tax-6* loss of function leads to significantly exacerbated TDP-43 Tg motor dysfunction and neurodegeneration [20]. To activate endogenous calcineurin in *C. elegans*, we employed a well-characterized constitutively active gain-of-function mutation [*tax-6*(*jh107)*], which has a deletion of the C-terminal calmodulin binding and autoinhibitory domain [60]. We crossed *tax-6(jh107)* animals with TDP-43 Tg animals and found TDP-43 Tg; *tax-6(jh107)* animals exhibited improved movement capabilities (Fig. 5a). We also tested whether increased levels of the calcineurin catalytic subunit would be sufficient to protect against TDP-43 driven motor impairment. To assess this, we generated transgenic *C. elegans* overexpressing (o/ex) wildtype *tax-6* (referred to as *tax-6* o/ex)*.* We found increased TAX-6 levels were also sufficient to improve TDP-43 Tg locomotion defects (Fig. 5b, Supplementary Fig. 7).

*C. elegans* have a stereotyped rhythmic feeding behavior, characterized by pharyngeal pumping to push their bacterial food source from the environment through a processing organ (i.e., grinder) into their intestine. Pharyngeal pumping represents coordinated signaling between well-defined neurons and muscles [61]. We tested whether constitutively active calcineurin/*tax-6(jh107)* could protect against TDP-43 driven pharyngeal pumping dysfunction using in vivo electrophysiology. To do this, we used a microfluidic chip-based system that records pharyngeal muscle and neuron action potentials extracellularly in individual animals [electropharyngeogram (EPG) recordings] [62]. We found TDP-43 Tg animals have a significant decrease in pumping frequency accompanied by an increased duration of pumping events (Fig. 5c–e). TDP-43 Tg; *tax-6(jh107)* animals were able to partially rescue pumping frequency and fully rescue pumping duration (Fig. 5c–e), indicating a restoration of neuronal function. Finally, we tested whether modulation of calcineurin activity could protect against neuronal loss observed in TDP-43 Tg *C. elegans.* We scored TDP-43 Tg; *tax-6(jh107)* animals for the presence of intact GABAergic motor neurons and found these animals lost fewer neurons relative to TDP-43 Tg alone (Fig. 5f). Therefore, activation of calcineurin is protective against TDP-43 Tg driven neuronal dysfunction and neurodegeneration in *C. elegans*.

## Discussion

TDP-43 phosphorylation is one of the most widely recognized pathological changes that occurs in TDP-43 proteinopathies, including ALS and FTLD-TDP [63, 64]. Model organism-based studies directly testing the role of TDP-43 phosphorylation have largely been limited to cell culture or invertebrate models and rely on phospho-mimetic or phospho-ablation techniques to examine consequences of phosphorylated TDP-43. Results from these studies have given contradictory information, where TDP-43 phospho-mimetics can promote TDP-43 nuclear localization or protect against TDP-43 aggregation, while TDP-43 phospho-ablation protects against TDP-43 neurotoxicity and subsequent neurodegeneration [21, 22, 65]. These differences may arise due to variations in cellular context, where differences in model systems or paradigms used to mimic or induce phosphorylated TDP-43 could impact its aggregation propensity. Small molecule inhibition of known TDP-43 kinases protects against neurodegeneration in mouse and cell culture models, supporting the idea that TDP-43 phosphorylation may be an adverse event for a healthy neuron [52, 66, 67].

In this study, we use an established mouse model of cytoplasmic TDP-43 mislocalization [34] and demonstrate robust depletion of the TDP-43 phosphatase calcineurin in multiple brain regions. Timing of calcineurin depletion coincides with increases in the TDP-43 kinase CDC7 and the appearance of phosphorylated TDP-43. These events precede neurodegeneration, which is robust only at late stages of disease in rNLS8 mice. We also demonstrate calcineurin activation reduces TDP-43 phosphorylation in mouse primary neurons and calcineurin activation or overexpression protects against TDP-43 neurotoxicity in *C. elegans* models of ALS/FTLD-TDP. Taken together, these data demonstrate brain responses to cytoplasmic TDP-43 that include changes in TDP-43 kinases and phosphatases regulating TDP-43 phosphorylation.

Reduced calcineurin activity has been observed in ALS patient lymphocytes and spinal cord [30, 31], suggesting calcineurin changes may also occur in human disease. In support of our study, decreased calcineurin protein has been shown in mouse primary cortical neurons or cortical brain lysate from mice overexpressing wildtype TDP-43 [29]. This study also found small interfering RNA (siRNA)-mediated reduction of TDP-43 in HEK293 cells led to increased calcineurin, suggesting calcineurin levels are responsive to TDP-43. TDP-43 is a major regulator of RNA metabolism events, including splicing. TDP-43 can regulate *Ppp3ca* mRNA exon exclusion [68] and *Ppp3ca, Ppp3cc,* and *Ppp3r1* polyadenylation (poly(A)) site selection [68, 69]. Poly(A) site selection impacts 3’ untranslated region (3’UTR) length controlling susceptibility to micro RNA (miRNA) binding and rates of translation, thus influencing transcripts and protein levels. It is possible this mechanism underlies the cell-type specific changes in mRNA and protein levels observed in rNLS8 mice. Our data suggest decreased calcineurin mRNA is an early event that occurs particularly in glutamatergic neurons. However, mechanisms by which this is controlled, and whether additional regulation of calcineurin levels exists at the transcriptional or post-translational levels, will require further study. Glutamatergic excitotoxicity has long been recognized as a feature of ALS [70]; changes in calcineurin could be contributory or a consequence of this.

We examine cell-type specific transcriptomic expression of TDP-43 kinases and phosphatases in the brain, at a time point when rNLS8 mice are strongly symptomatic, but preceding major neuronal loss (4 weeks post-induction). From this, we find the TDP-43 phosphatase calcineurin is downregulated, while the TDP-43 phosphatase PP1 is upregulated, largely in subsets of glutamatergic neurons. We also find changes in the TDP-43 kinases Cdc7, CK1α, Mapk11, Ttbk1, and Ttbk2 in a small number of cell clusters. We do not yet know why regulatory differences in kinase and phosphatase transcription exist in select cell subtypes, but these differences in cellular responses to cytoplasmic TDP-43 may contribute to cell-type and brain region differences in resistance or resilience to ALS/FTLD-TDP. For kinases and phosphatases that do not exhibit transcription or protein level changes in response to cytoplasmic TDP-43, there may still be activity level regulation that contributes to accumulation or clearance of pathologic TDP-43. Therefore, additional dissection of these pathways controlling TDP-43 phosphorylation are warranted.

Calcineurin is best known for its role in promoting cellular immune responses, including thymus cell (T cell) activation [71]. Clinically, calcineurin inhibitors have been used for more than 40 years as immunosuppressant drugs to prevent solid organ transplant rejection or treat autoimmune disorders [72]. Calcineurin reduction or inhibition in models of TDP-43 proteinopathy increases accumulation of phosphorylated TDP-43 and worsens disease phenotypes [20]. However, clinical use of calcineurin inhibitors has not been linked to development of TDP-43 proteinopathies. In contrast, use of the brain penetrant calcineurin inhibitor tacrolimus is associated with decreased rates of Alzheimer’s disease dementia and Parkinson’s disease [73, 74]. For patients with ALS and FTLD-TDP, we would predict inhibition of calcineurin would promote TDP-43 phosphorylation and worsen disease. In fact, the calcineurin inhibitor tacrolimus was included as part of a cocktail of immunosuppressive drugs used in a 6 month open-label Phase 2 trial testing whether reduced immune responses would be protective in patients with ALS [27]. Nearly half of the 31 patients enrolled failed to complete the study, and no benefit was shown from the treatment. While the reasons for the failure of this trial remain unknown, inclusion of tacrolimus as part of the treatment may have worsened TDP-43 pathology and thus masked protective effects of immunosuppression. Our data demonstrate activation of calcineurin reduces phosphorylated TDP-43 and restores neuronal homeostasis in models of ALS and FTLD-TDP. Based on these results, calcineurin may be a critical contributor to resilience against phosphorylated TDP-43, and methods to activate or increase calcineurin should be developed as potential therapeutic strategies.

## Conclusions

Our study provides a temporal map of calcineurin changes in the brain occurring during disease progression in a mouse model of ALS/FTLD-TDP. These changes occur downstream of the disease-triggering event, TDP-43 mislocalization to the cytoplasm, and they likely reinforce accumulation of phosphorylated TDP-43. We demonstrate increasing calcineurin activity restores homeostasis of TDP-43 phosphorylation and protects against neurotoxic phenotypes in model systems. Kinase and phosphatase dysregulation events precede neurodegeneration, making restoration of these brain resilience pathways compelling interventions with the potential to modify phosphorylated TDP-43. Taken together, these targets could broadly protect against TDP-43 proteinopathies where TDP-43 hyperphosphorylation is present, including ALS and FTLD-TDP.

## Supplementary Information

Below is the link to the electronic supplementary material.

Supplementary Material 1. Figure S1: Phosphorylated TDP-43 in rNLS8 mouse brain appears at symptomatic timepoints. Representative images of phosphorylated TDP-43 immunostaining in control animals, and rNLS8 animals at 1 week, 4 weeks, and 7 weeks post-induction. **a**–**d** hippocampus, **e**–**h** motor cortex, and **i**–**l** dorsal striatum. No phosphorylated TDP-43 was detected in control animals or in rNLS8 animals at 1 week post-induction. Arrows point to representative phosphorylated TDP-43 inclusions. Scale bars: 25 µm.

Supplementary Material 2. Figure S2: Long exposure of immunoblot detecting endogenous TDP-43 present in rNLS8 mouse brain. Overexposure of representative immunoblots from Fig. 2 showing expression of endogenous TDP-43 in control animals and overexpression of endogenous and human TDP-43 in rNLS8 animals at 1 week, 4 weeks, and 7 weeks post-induction. **a** hippocampus, **b** cortex, and **c** striatum.

Supplementary Material 3. Figure S3: Hippocampal neurodegeneration in end-stage rNLS8 mouse brain. **a**–**d** Representative images of neuronal marker Rbfox3 (NeuN) immunostaining in the hippocampus CA3 region in control animals, and rNLS8 animals at 1 week, 4 weeks, and 7 weeks post-induction. Scale bars: 100 µm. Neuronal density was assessed by measuring NeuN immunoreactivity (**e**–**g**) and NeuN positive cells (**h**–**j**) in CA3 hippocampal region. Statistical analysis is by unpaired *t*-test, two-tailed [*n* = 8 control, 15 rNLS8 animals (1 week); 8 control, 16 rNLS8 animals (4 weeks); 15 control, 15 rNLS8 animals (7 weeks); ns: *p* > 0.05, **: *p* < 0.01, ****: *p* ≤ 0.0001]. All bar graphs represent mean ± SEM.

Supplementary Material 4. Figure S4: Cortical atrophy in symptomatic and end-stage rNLS8 mouse brain. Representative images of neuronal marker Rbfox3 (NeuN) immunostaining in control animals, and rNLS8 animals at 1 week, 4 weeks, and 7 weeks post-induction (**a**–**d; h**–**k**). Scale bars: 500 µm. Cortical atrophy was assessed by measuring thickness of the motor cortex region (**a**–**g**, vertical bar in **a**–**d** showing location of measurement) and cingulate cortex region (**h**–**n**, vertical bar in **h**–**k** showing location of measurement), as well as cortical area (**o**–**q**). Statistical analysis is by unpaired *t*-test, two-tailed [*n* = 8 control, 15 rNLS8 animals (1 week); 8 control, 16 rNLS8 animals (4 weeks); 15 control, 15 rNLS8 animals (7 weeks); ns: *p* > 0.05, *: *p* < 0.05, **: *p* ≤ 0.01, ****: *p* ≤ 0.0001]. All bar graphs represent mean ± SEM.

Supplementary Material 5. Figure S5: Differential gene expression changes and gene ontology in a glutamatergic neuron cell population in symptomatic rNLS8 mouse brain. Differential gene expression in layer 4/5 intratelencephalically projecting neurons. **a** Volcano plot showing significant gene expression changes. **b** Gene ontology analysis highlighting the most impacted pathways by significant gene upregulation or downregulation.

Supplementary Material 6. Figure S6: Primary neuron controls and full immunoblots. **a**–**c** Mouse primary neurons without exposure to EA have very low or no apparent phosphorylated TDP-43. Arrowhead is at approximate location of full-length TDP-43. **d** Representative immunostaining of primary neurons for MAPT (red), and DAPI (blue). Scale bar: 25 µm. **e**–**h** Full immunoblots for quadruplicate immunoblot experiments presented in Fig. 4. Arrowhead is at approximate location of full-length TDP-43.

Supplementary Material 7. Figure S7: Activation of calcineurin using an independent transgene protects against TDP-43 neurotoxicity in *C. elegans*. **a** A second transgene overexpressing wildtype TAX-6 in *C. elegans* (*tax-6 o/ex 2*) improves TDP-43 Tg motor dysfunction. Statistical analysis is by unpaired* t*-test, two-tailed (*n* ≥ 150; *N* = 3 independent experiments; *: *p* < 0.05).

Supplementary Material 8. Full immunoblots for all figures

Supplementary Material 9. Table S1: A list of significantly (*p adj* < 0.05, and log >|1.0|) differentially expressed genes listed by cell type class and regulation (upregulation or downregulation).

Supplementary Material 10. Table S2: Gene ontology for layer 4/5 intratelencephalically projecting neurons in symptomatic rNLS8 mouse brain. Tab 1 (DE Genes) lists differential gene expression analysis for this cluster. Tab 2 (DAVID GO Results) lists overrepresented gene ontology terms from an analysis for percent enrichment of biological processes of significantly upregulated or downregulated genes.

Supplementary Material 11. Table S3: Survey of TDP-43 kinases and phosphatases using TDP-map proteomics dataset. Table 1 lists results for kinases and phosphatases detected in the dataset in both summary format (down, no change, up), and fold change (FC) and p-value (Pval) data for 1, 2, 4, and 6 weeks post-induction (off Dox) and at 6 weeks post-induction (off Dox) + 2 weeks of recovery (on Dox). Table 2 lists the kinases and phosphatases surveyed.

## Data Availability

The data analyzed for this study are published in this manuscript and associated supplementary information. In addition, snRNAseq data is available at GEO accession GSE303077.

## Abbreviations

3’UTR: 3’ untranslated region
AAALAC: American Association for Accreditation of Laboratory Animal Care
AD: Alzheimer’s disease
ALS: Amyotrophic lateral sclerosis
CA1-Pros: Hippocampal CA1-prosubiculum glutamatergic neurons
CaN: Calcineurin
CB Gran: Cerebellar granule glutamatergic neurons
CDC7: Cell division cycle 7-related protein kinase
cDNA: Complementary DNA
cds: Cell data set
C. elegans: *Caenorhabditis elegans*
CK1: Casein kinase 1
CTE: Chronic traumatic encephalopathy
DAB: Diaminobenzidine
DAVID: Database for annotation, visualization, and integrated discovery
Dorsal striatum: Caudate nucleus and putamen
DOX: Doxycycline
EA: Ethacrynic acid
EPG: Electropharyngeogram
FTLD-TDP: Frontotemporal lobar degeneration with TDP-43 pathology
GABA: Gamma-aminobutyric acid
GFP: Green fluorescent protein
GO: Gene ontology
HD: Huntington’s disease
hTDP-43ΔNLS: Mutant human TDP-43 with deletion of nuclear localization signal
IACUC: Institutional animal care and use committee
IHC: Immunohistochemistry
IKKβ: Inhibitory kappa B kinase beta
IR: Immunoreactivity
L6 CT: Cortical layer 6 corticothalamic-projecting glutamatergic neurons
L2/3 IT: Cortical layer 2/3 intratelencephalic-projecting glutamatergic neurons
L4/5 IT: Cortical layer 4/5 intratelencephalic-projecting glutamatergic neurons
L6 IT: Cortical layer 6 intratelencephalic-projecting glutamatergic neurons
LATE: Limbic-predominant age-related TDP-43 encephalopathy
LBD: Lewy body dementia
miRNA: Micro RNA
mRNA: Messenger RNA
NB-A: Neurobasal A
NEFH: Neurofilament heavy polypeptide
NeuN: Neuronal marker Rbfox3
NGM: Nematode growth media
NiCl_2_: Nickel chloride
NLS: Nuclear localization signal
non-Tg: Non-transgenic
OD: Optical density
o/ex: Overexpression
p38α: Mitogen-activated protein kinase 14
PCA: Principal components analysis
poly(A): Polyadenylation
PP1: Protein phosphatase 1
Proteinopathy: Protein misfolding disease
pTDP-43: Phosphorylated TDP-43
qRT-PCR: Quantitative reverse transcription PCR
rNLS8: Inducible mouse model of ALS and FTLD-TDP
sci-RNA-seq3: Single-nucleus combinatorial indexing RNA-seq
siRNA: Small interfering RNA
snRNAseq: Single nucleus RNA sequencing
*tax-6*(*jh107)*: Mutant *tax-6* gain-of-function *C. elegans* strain
*tax-6* o/ex: Wildtype *tax-6* overexpression transgenic *C. elegans* models
T cell: Thymus cell
TDP-43: Transactive response DNA binding protein of 43 kDa
TDP-43 Tg: Human mutant TDP-43 transgenic *C. elegans* model
TTBK1: Tau tubulin kinase 1
TTBK2: Tau tubulin kinase 2
UMAP: Uniform manifold approximation and projection
WT: Control animals
