# Supplementary figures and images for: Calcineurin depletion coincides with phosphorylated TDP-43 deposition in a mouse model of ALS/FTLD-TDP
Source: Acta Neuropathol Commun. 2026 Jan 3;14:33. doi: 10.1186/s40478-025-02192-9 (PMC12870385; doi:10.1186/s40478-025-02192-9)

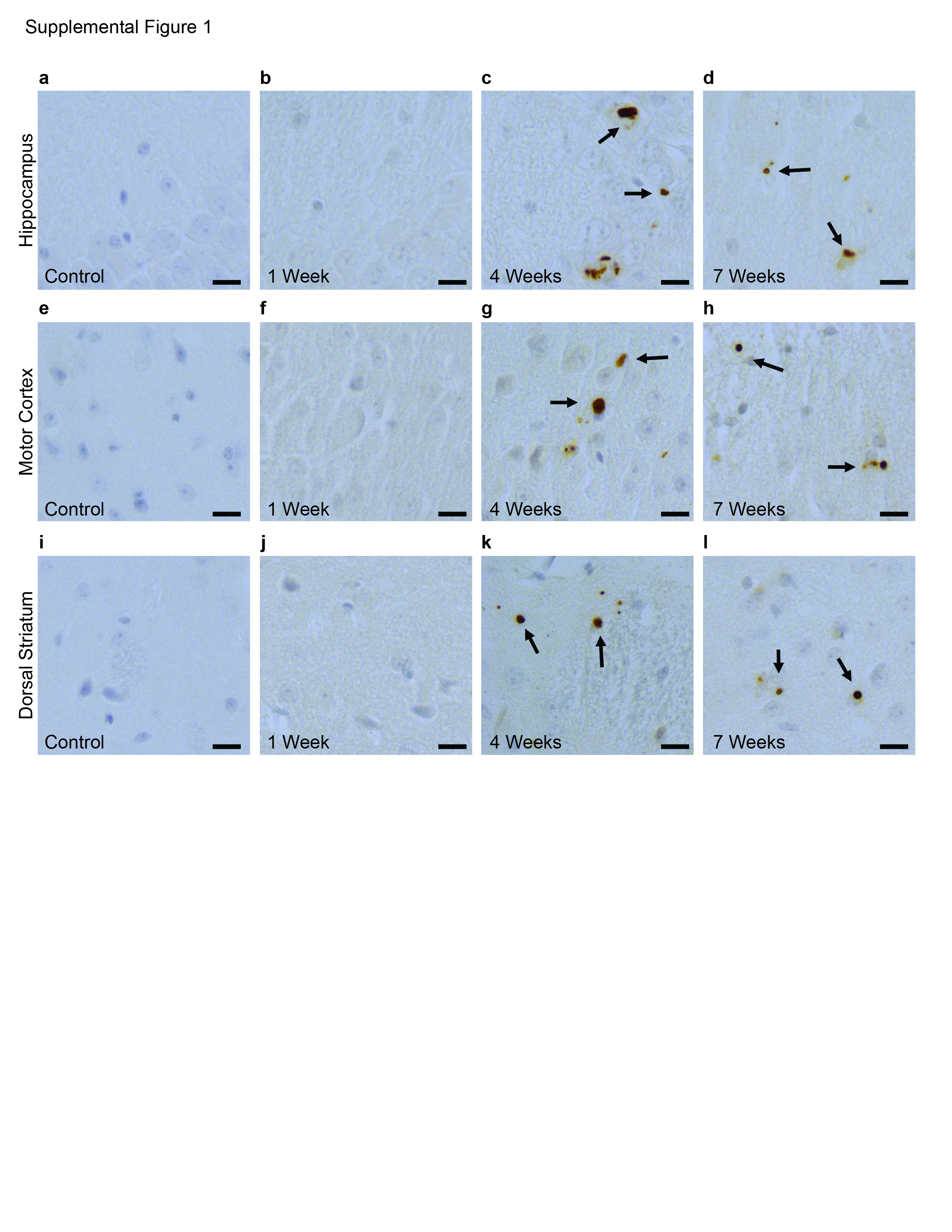

Supplement: Supplementary file 1 — Supplementary Material 1. Figure S1: Phosphorylated TDP-43 in rNLS8 mouse brain appears at symptomatic timepoints. Representative images of phosphorylated TDP-43 immunostaining in control animals, and rNLS8 animals at 1 week, 4 weeks, and 7 weeks post-induction. a–d hippocampus, e–h motor cortex, and i–l dorsal striatum. No phosphorylated TDP-43 was detected in control animals or in rNLS8 animals at 1 week post-induction. Arrows point to representative phosphorylated TDP-43 inclusions. Scale bars: 25 µm. [file 40478_2025_2192_MOESM1_ESM.tif]

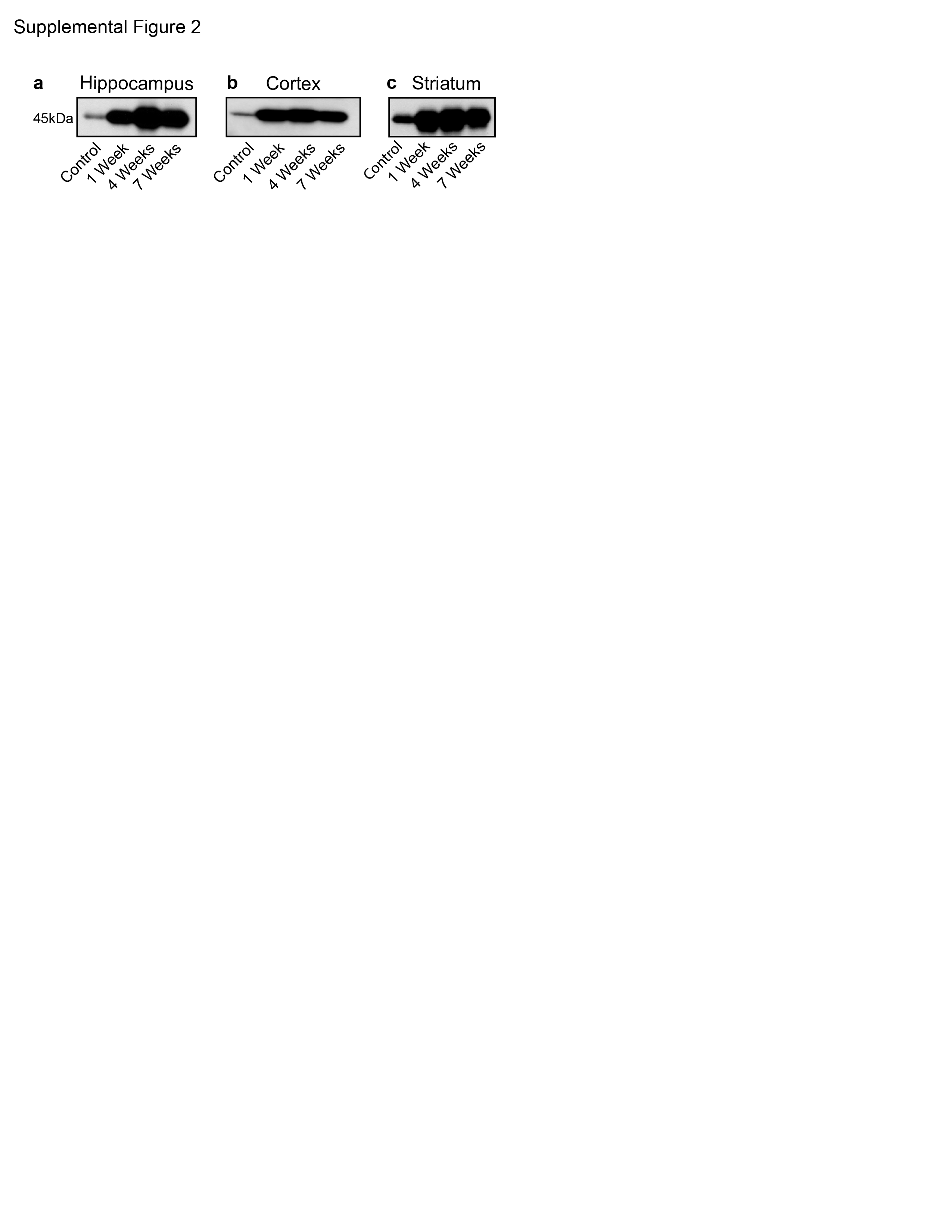

Supplement: Supplementary file 2 — Supplementary Material 2. Figure S2: Long exposure of immunoblot detecting endogenous TDP-43 present in rNLS8 mouse brain. Overexposure of representative immunoblots from Fig. 2 showing expression of endogenous TDP-43 in control animals and overexpression of endogenous and human TDP-43 in rNLS8 animals at 1 week, 4 weeks, and 7 weeks post-induction. a hippocampus, b cortex, and c striatum. [file 40478_2025_2192_MOESM2_ESM.tif]

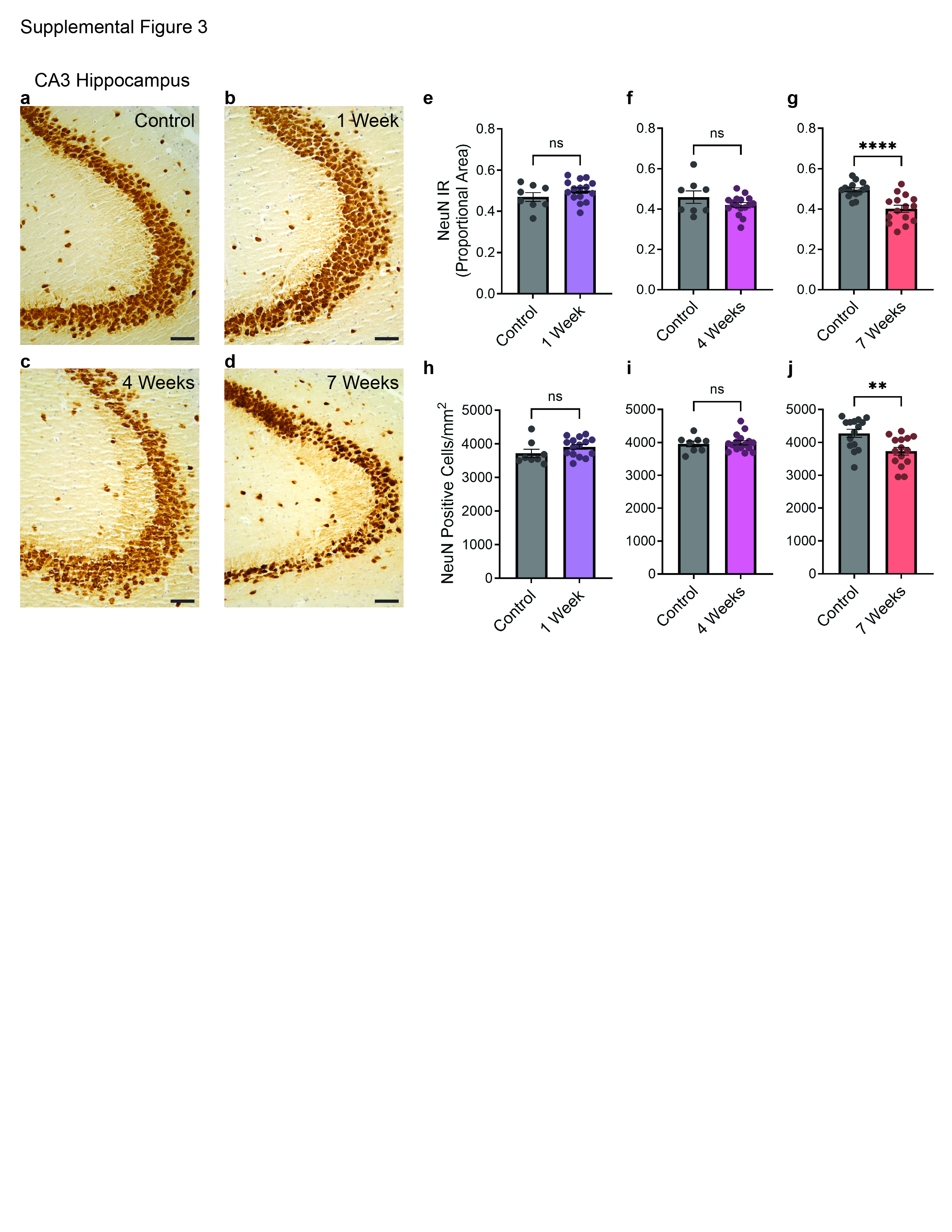

Supplement: Supplementary file 3 — Supplementary Material 3. Figure S3: Hippocampal neurodegeneration in end-stage rNLS8 mouse brain. a–d Representative images of neuronal marker Rbfox3 (NeuN) immunostaining in the hippocampus CA3 region in control animals, and rNLS8 animals at 1 week, 4 weeks, and 7 weeks post-induction. Scale bars: 100 µm. Neuronal density was assessed by measuring NeuN immunoreactivity (e–g) and NeuN positive cells (h–j) in CA3 hippocampal region. Statistical analysis is by unpaired t-test, two-tailed [n = 8 control, 15 rNLS8 animals (1 week); 8 control, 16 rNLS8 animals (4 weeks); 15 control, 15 rNLS8 animals (7 weeks); ns: p > 0.05, **: p < 0.01, ****: p ≤ 0.0001]. All bar graphs represent mean ± SEM. [file 40478_2025_2192_MOESM3_ESM.tif]

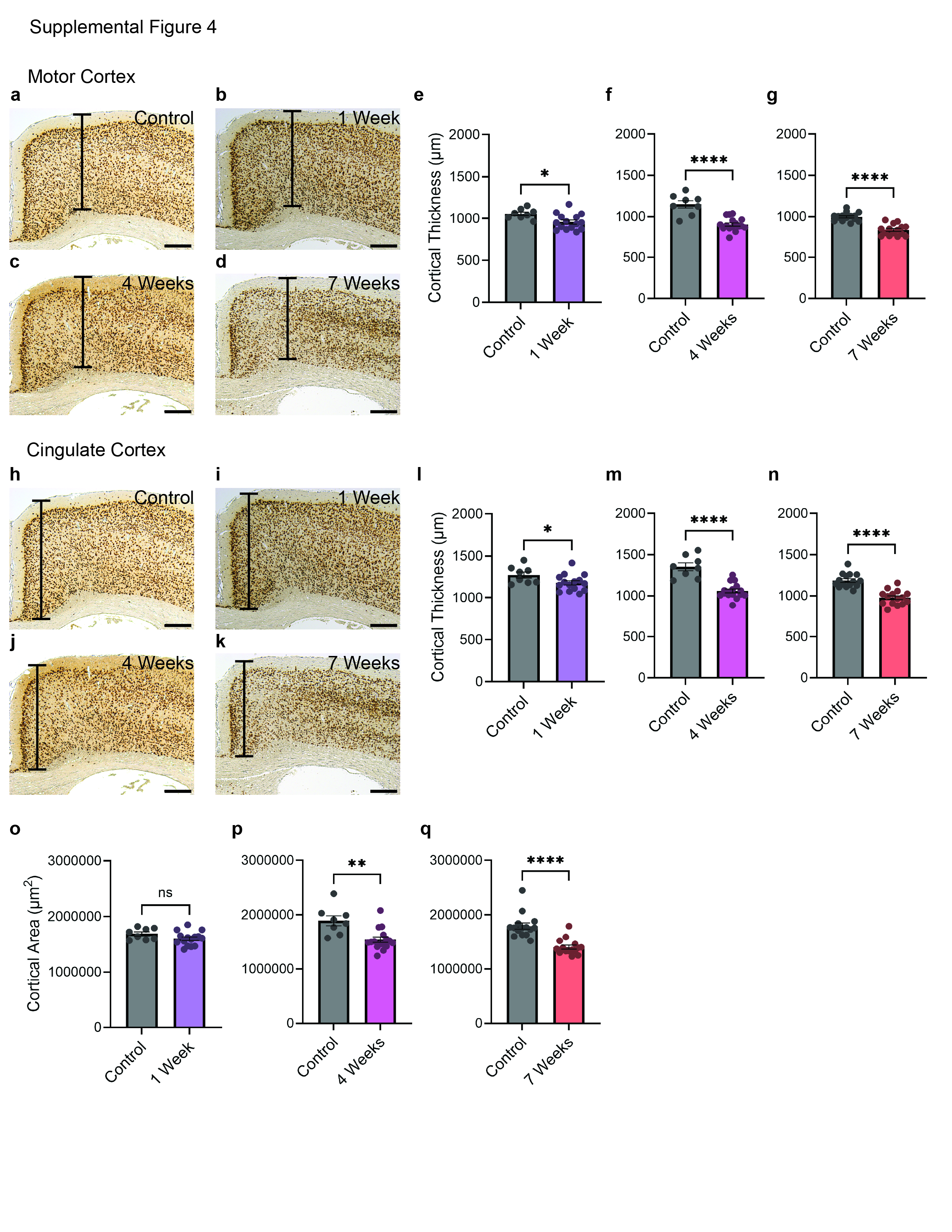

Supplement: Supplementary file 4 — Supplementary Material 4. Figure S4: Cortical atrophy in symptomatic and end-stage rNLS8 mouse brain. Representative images of neuronal marker Rbfox3 (NeuN) immunostaining in control animals, and rNLS8 animals at 1 week, 4 weeks, and 7 weeks post-induction (a–d; h–k). Scale bars: 500 µm. Cortical atrophy was assessed by measuring thickness of the motor cortex region (a–g, vertical bar in a–d showing location of measurement) and cingulate cortex region (h–n, vertical bar in h–k showing location of measurement), as well as cortical area (o–q). Statistical analysis is by unpaired t-test, two-tailed [n = 8 control, 15 rNLS8 animals (1 week); 8 control, 16 rNLS8 animals (4 weeks); 15 control, 15 rNLS8 animals (7 weeks); ns: p > 0.05, *: p < 0.05, **: p ≤ 0.01, ****: p ≤ 0.0001]. All bar graphs represent mean ± SEM. [file 40478_2025_2192_MOESM4_ESM.tif]

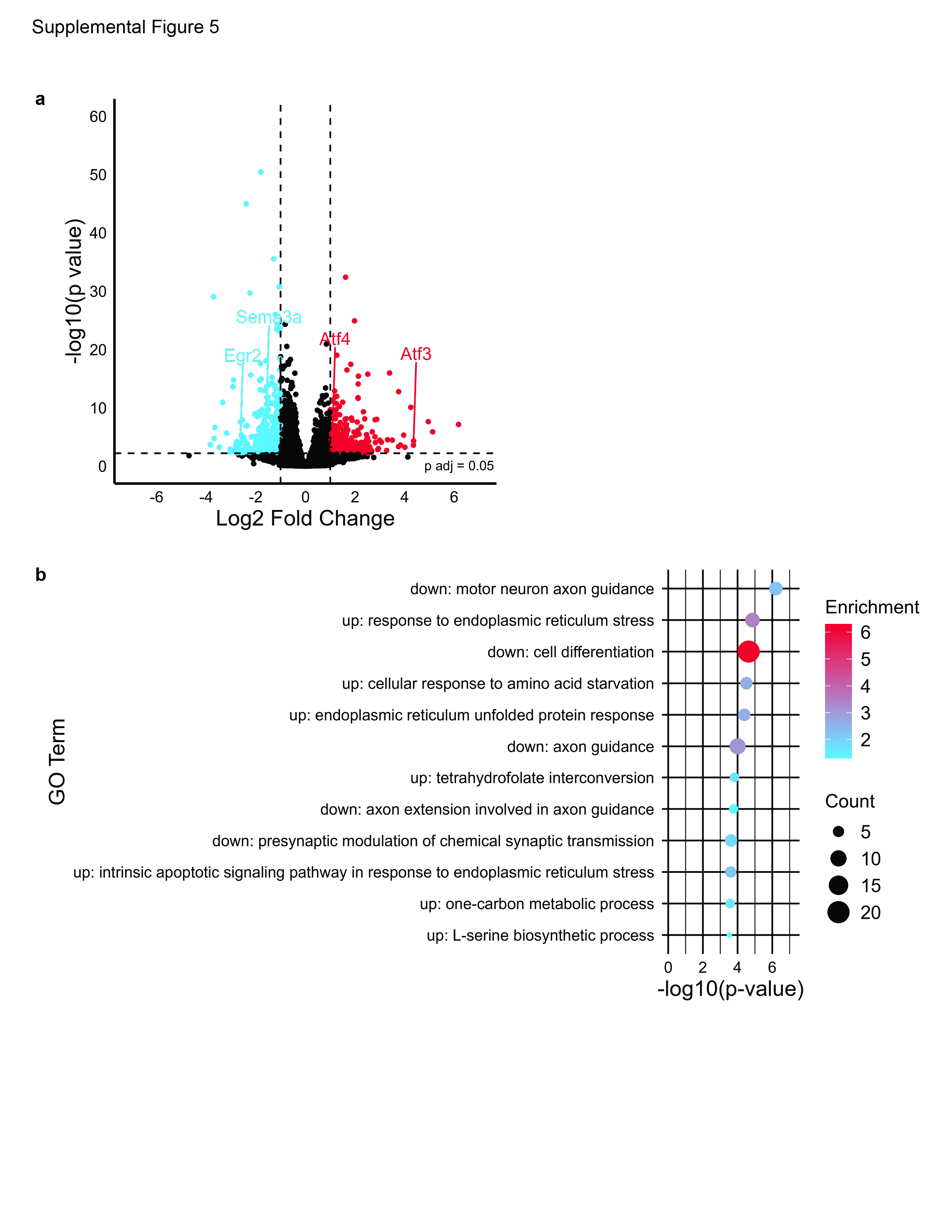

Supplement: Supplementary file 5 — Supplementary Material 5. Figure S5: Differential gene expression changes and gene ontology in a glutamatergic neuron cell population in symptomatic rNLS8 mouse brain. Differential gene expression in layer 4/5 intratelencephalically projecting neurons. a Volcano plot showing significant gene expression changes. b Gene ontology analysis highlighting the most impacted pathways by significant gene upregulation or downregulation. [file 40478_2025_2192_MOESM5_ESM.tif]

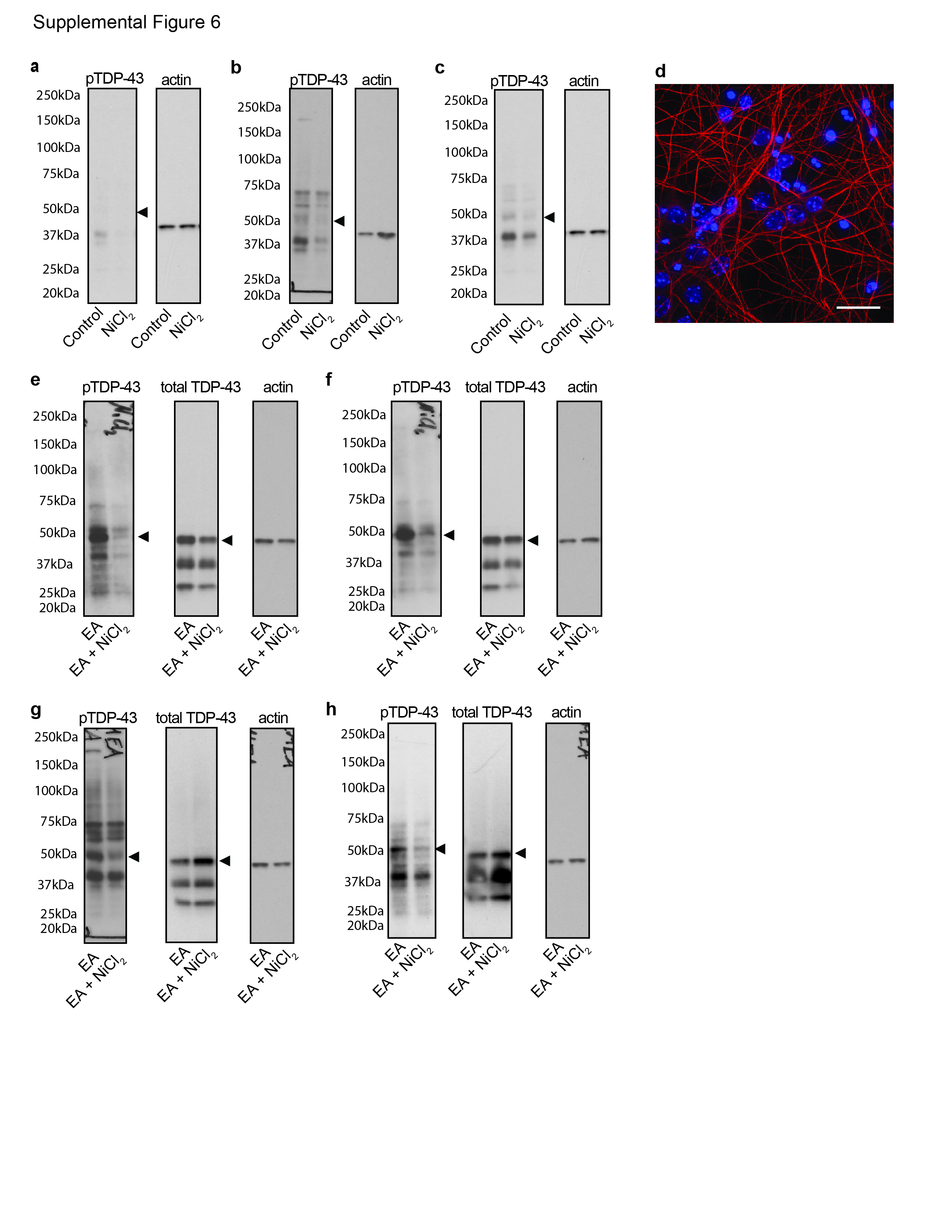

Supplement: Supplementary file 6 — Supplementary Material 6. Figure S6: Primary neuron controls and full immunoblots. a–c Mouse primary neurons without exposure to EA have very low or no apparent phosphorylated TDP-43. Arrowhead is at approximate location of full-length TDP-43. d Representative immunostaining of primary neurons for MAPT (red), and DAPI (blue). Scale bar: 25 µm. e–h Full immunoblots for quadruplicate immunoblot experiments presented in Fig. 4. Arrowhead is at approximate location of full-length TDP-43. [file 40478_2025_2192_MOESM6_ESM.tif]

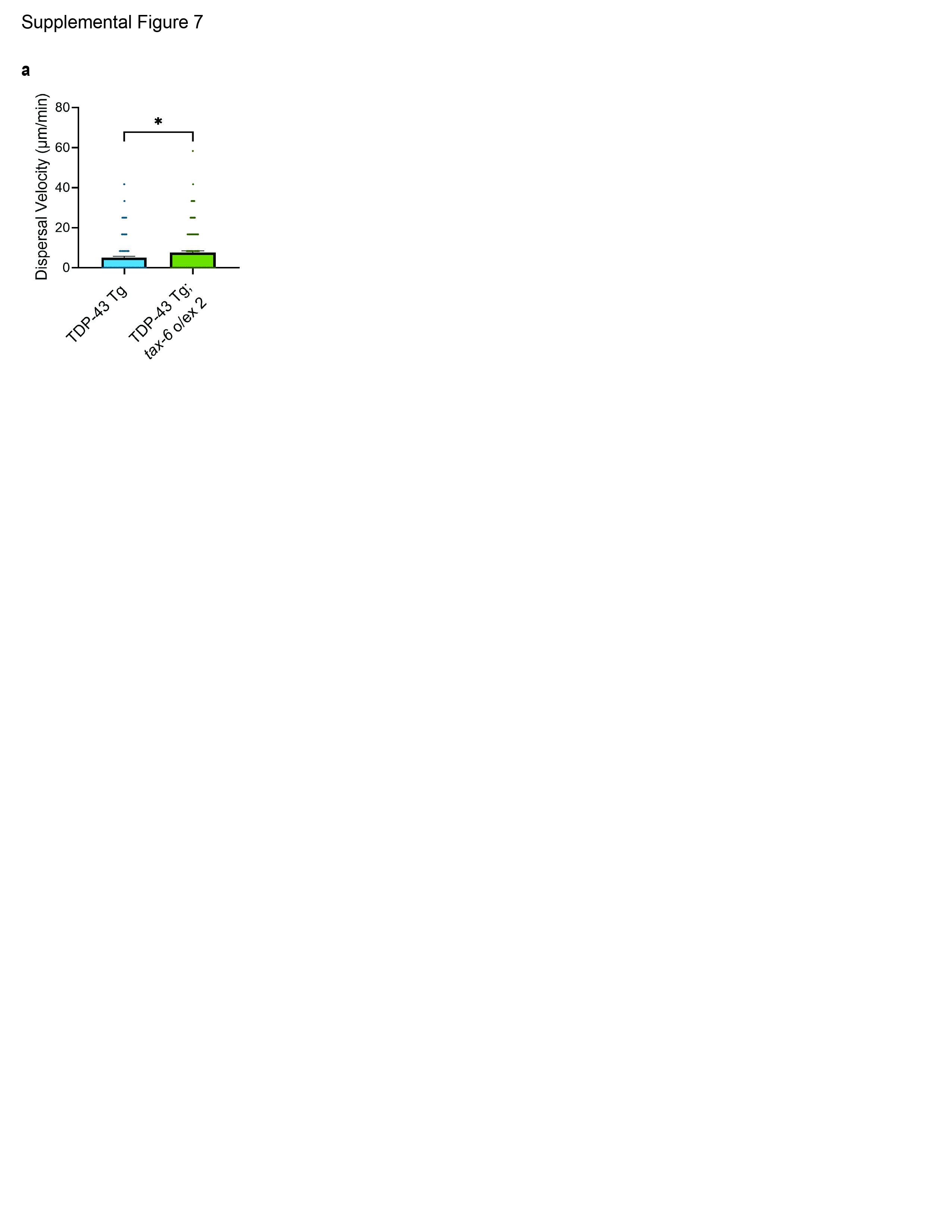

Supplement: Supplementary file 7 — Supplementary Material 7. Figure S7: Activation of calcineurin using an independent transgene protects against TDP-43 neurotoxicity in C. elegans. a A second transgene overexpressing wildtype TAX-6 in C. elegans (tax-6 o/ex 2) improves TDP-43 Tg motor dysfunction. Statistical analysis is by unpaired t-test, two-tailed (n ≥ 150; N = 3 independent experiments; *: p < 0.05). [file 40478_2025_2192_MOESM7_ESM.tif]
