# Supplementary material for: Calcineurin depletion coincides with phosphorylated TDP-43 deposition in a mouse model of ALS/FTLD-TDP
Source: Acta Neuropathol Commun. 2026 Jan 3;14:33. doi: 10.1186/s40478-025-02192-9 (PMC12870385; doi:10.1186/s40478-025-02192-9)

## Fig 2A and Supplemental Fig 2: Hippocampus

actin

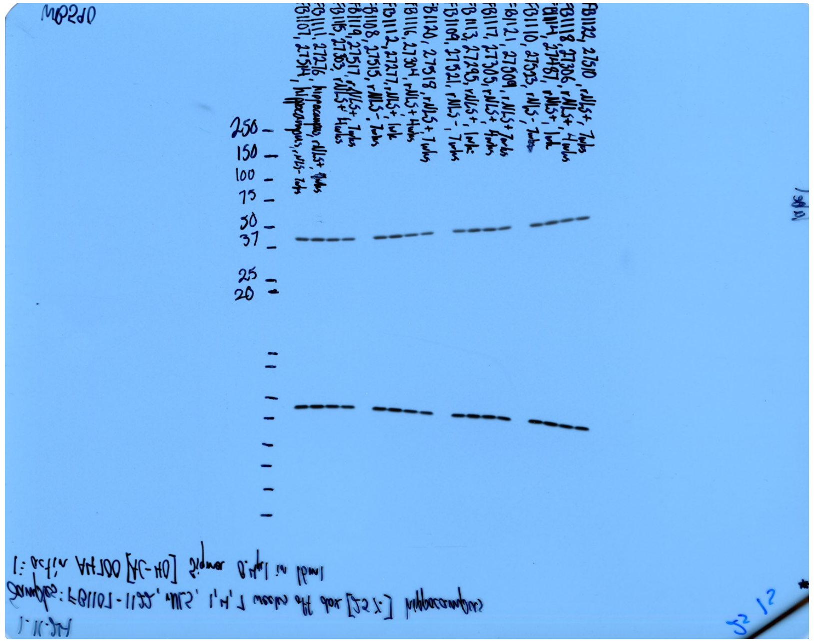

pTDP-43

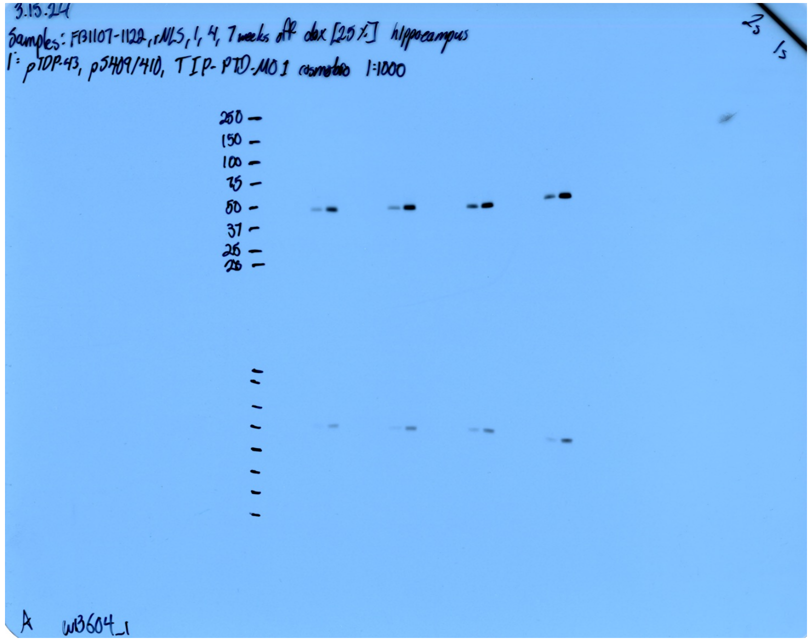

total TDP-43

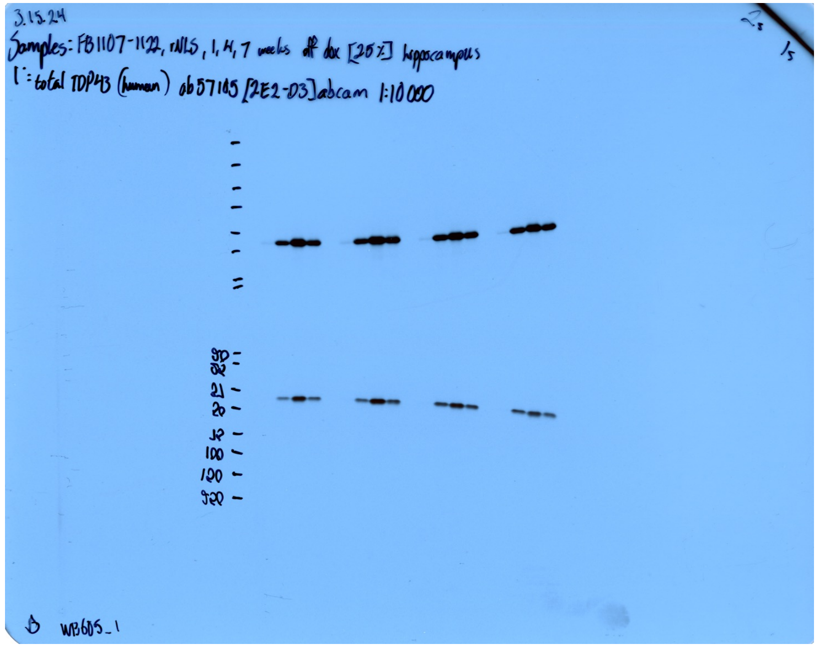

calcineurin

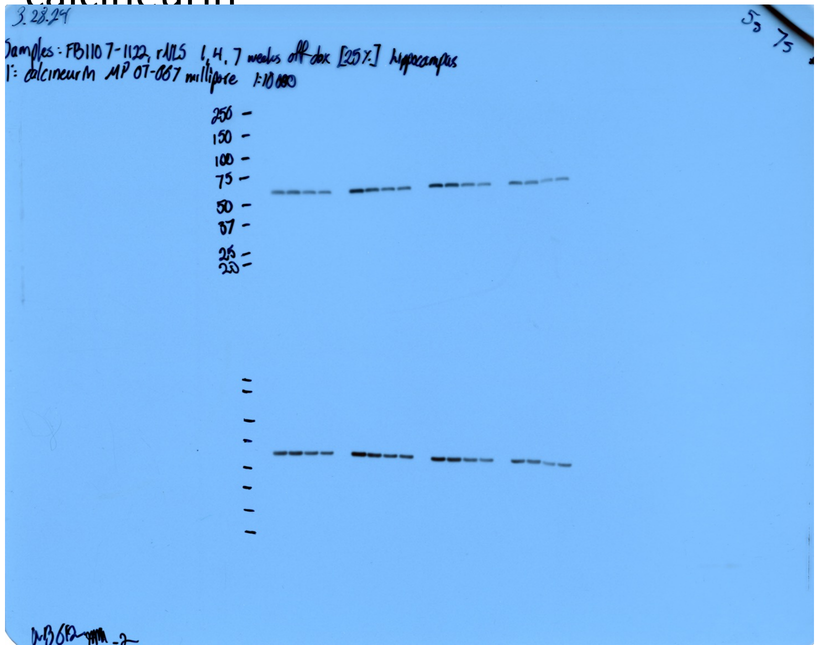

CDC7

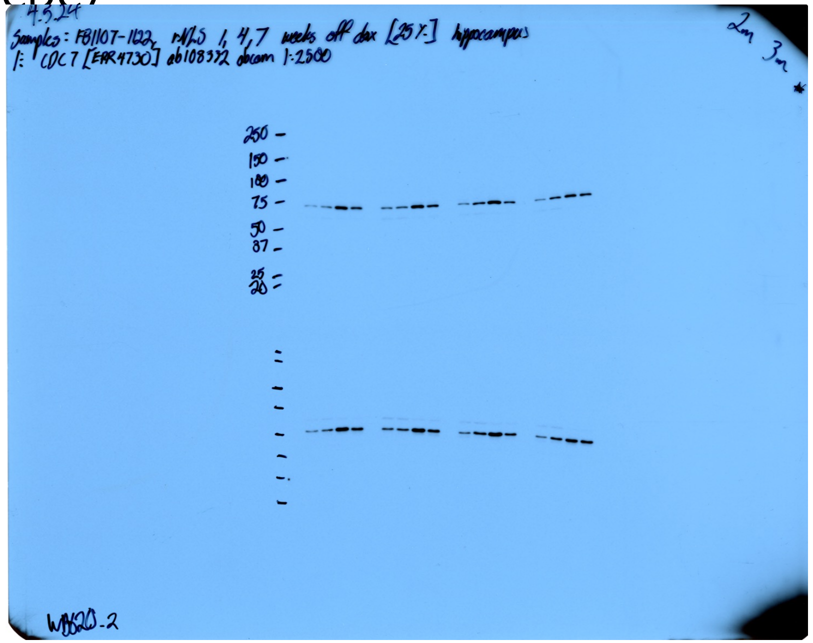

total TDP-43

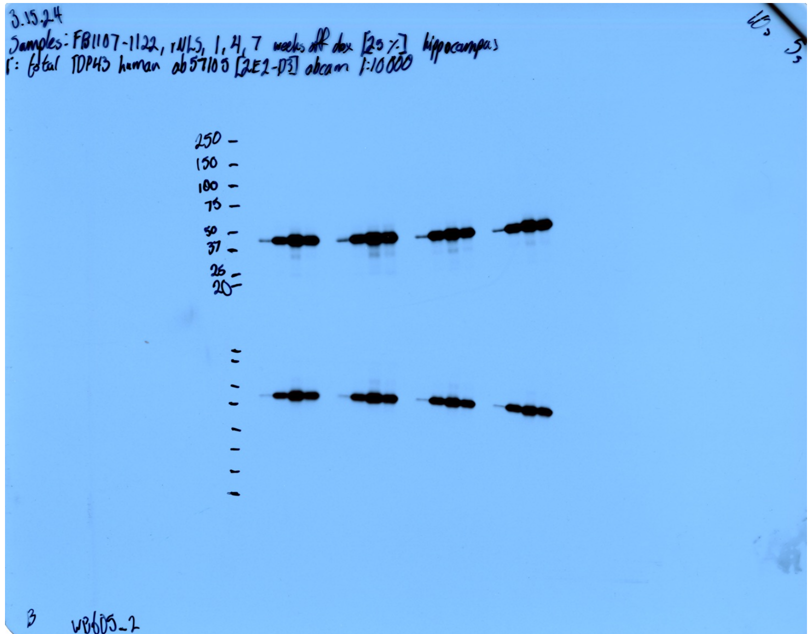

**Fig 2F and Supplemental Fig 2: Cortex**

actin

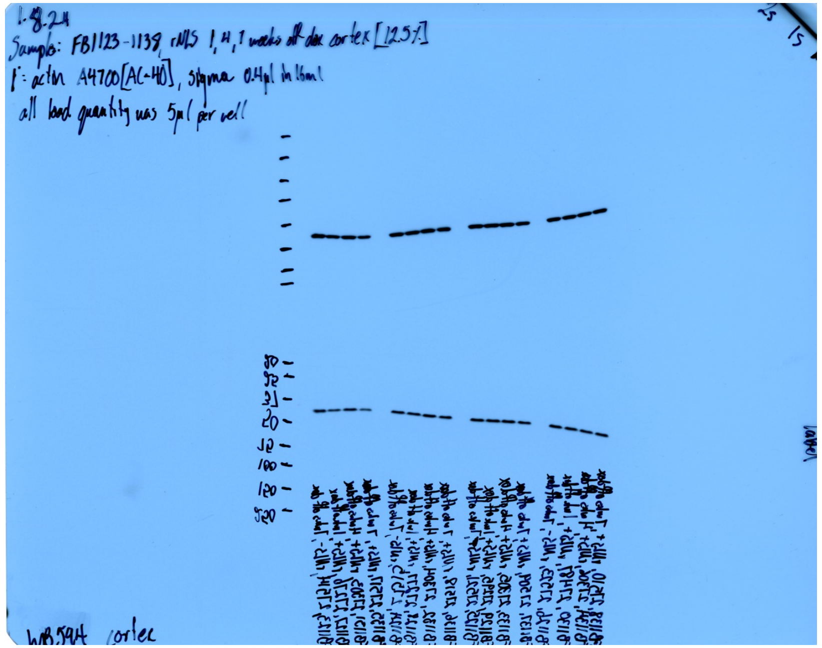

pTDP-43

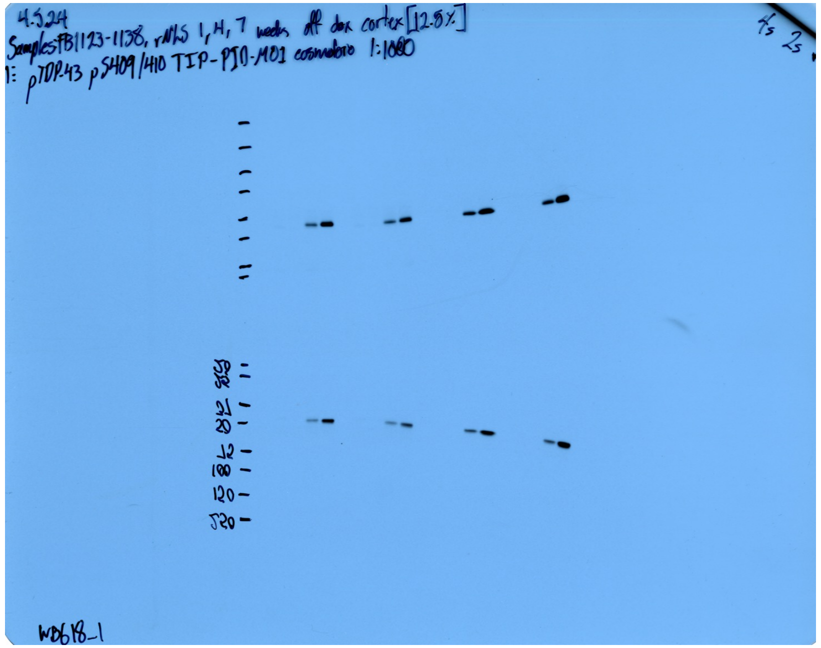

total TDP-43

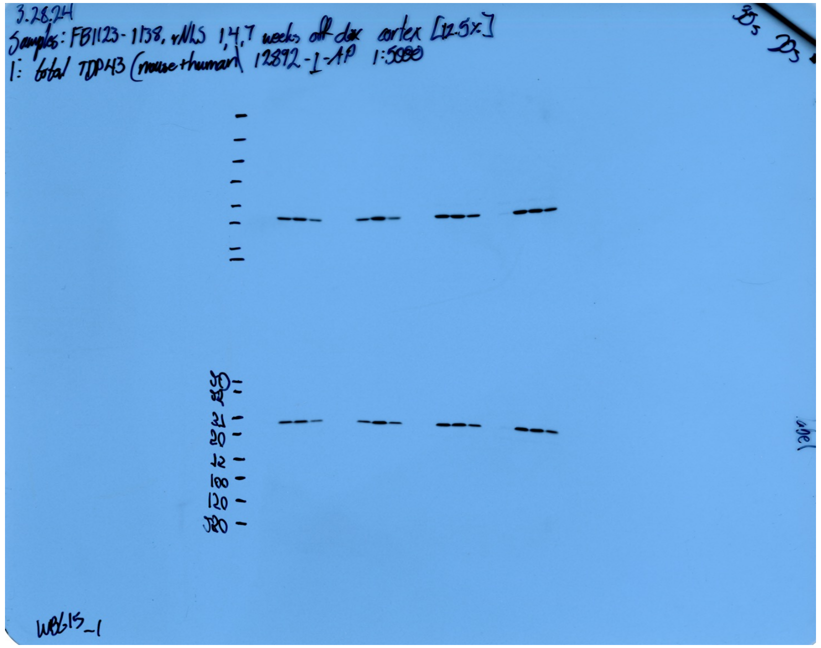

calcineurin

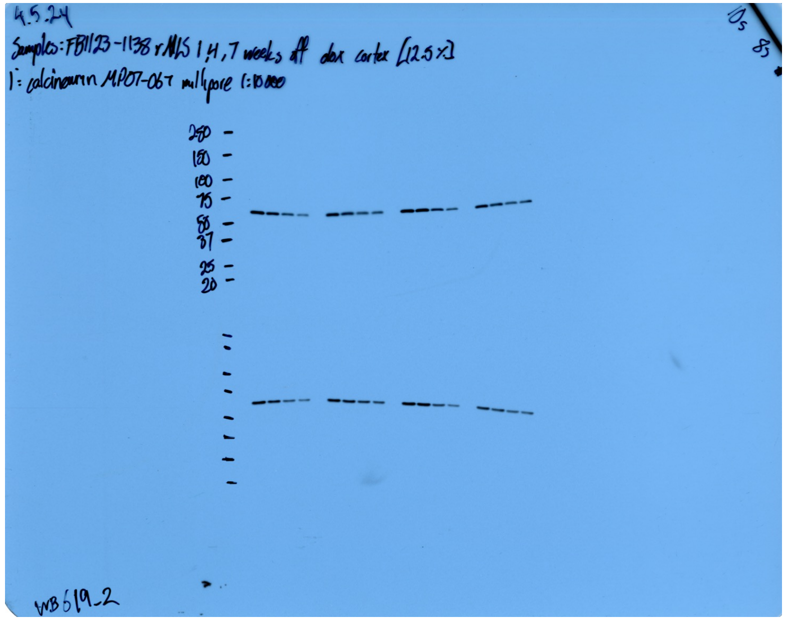

CDC7

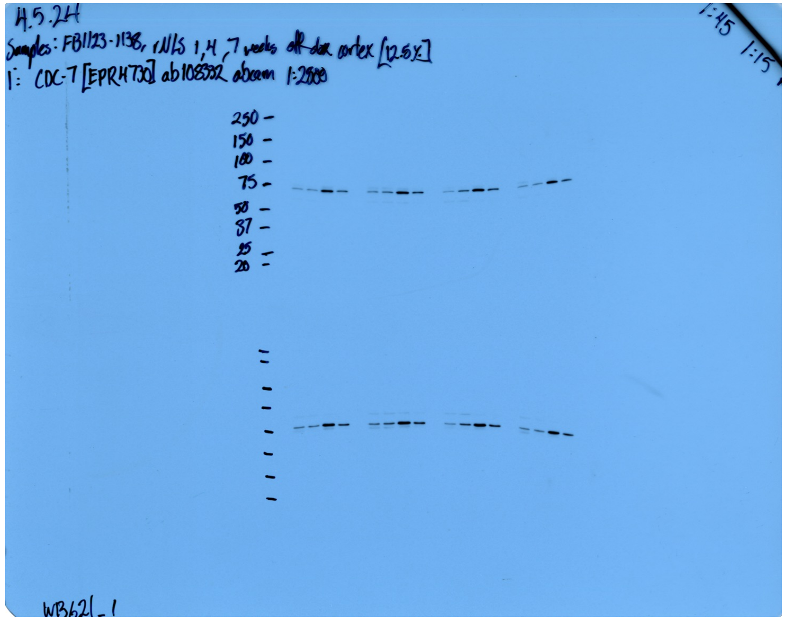

total TDP-43

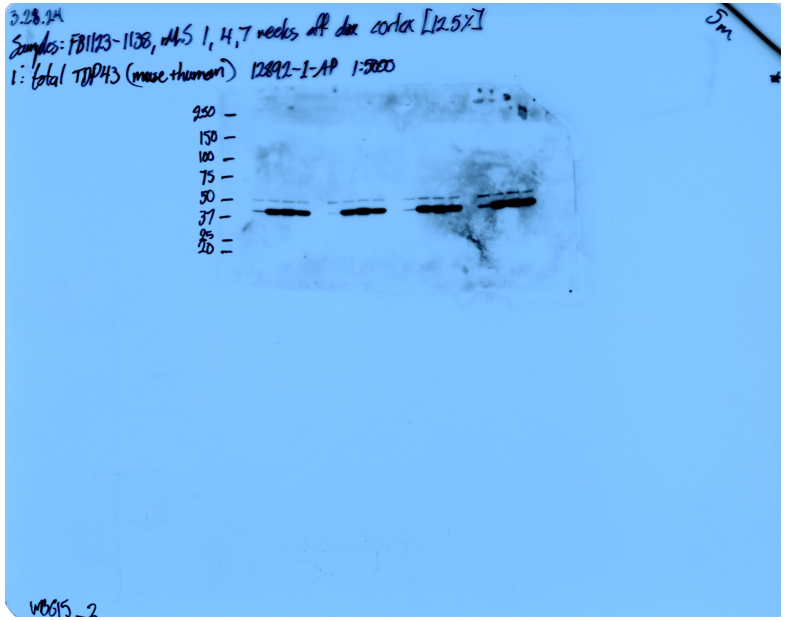

# Fig 2K and Supplemental Fig 2: Striatum

actin

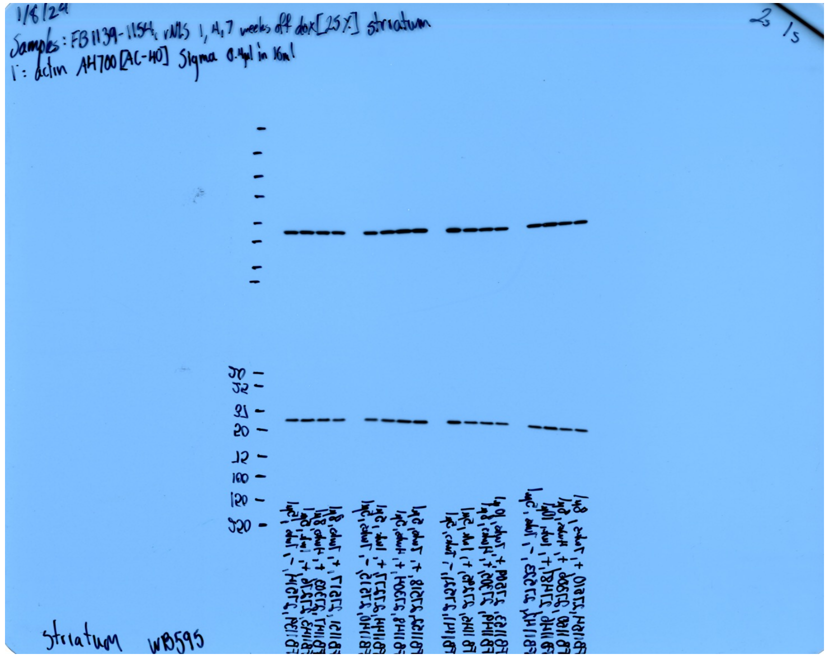


pTDP-43

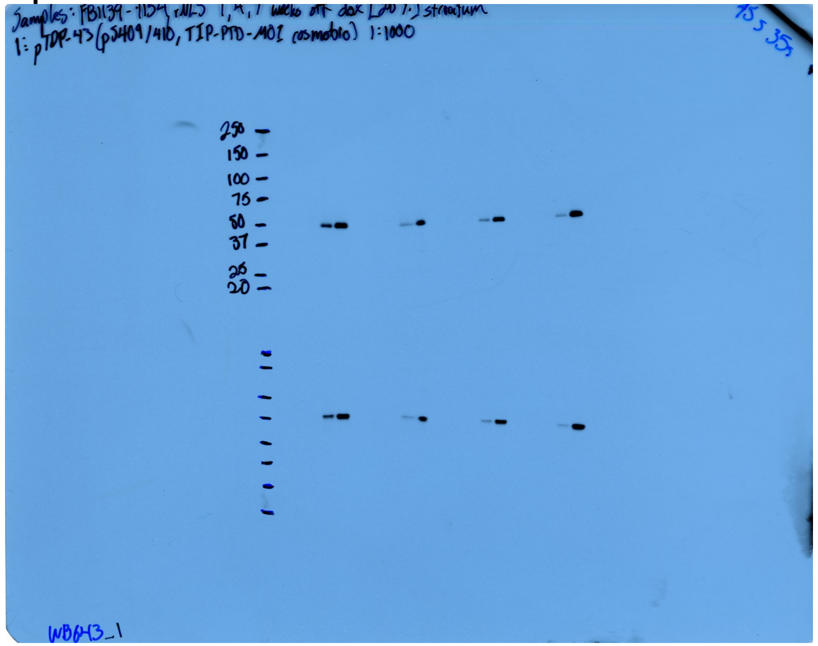


total TDP-43

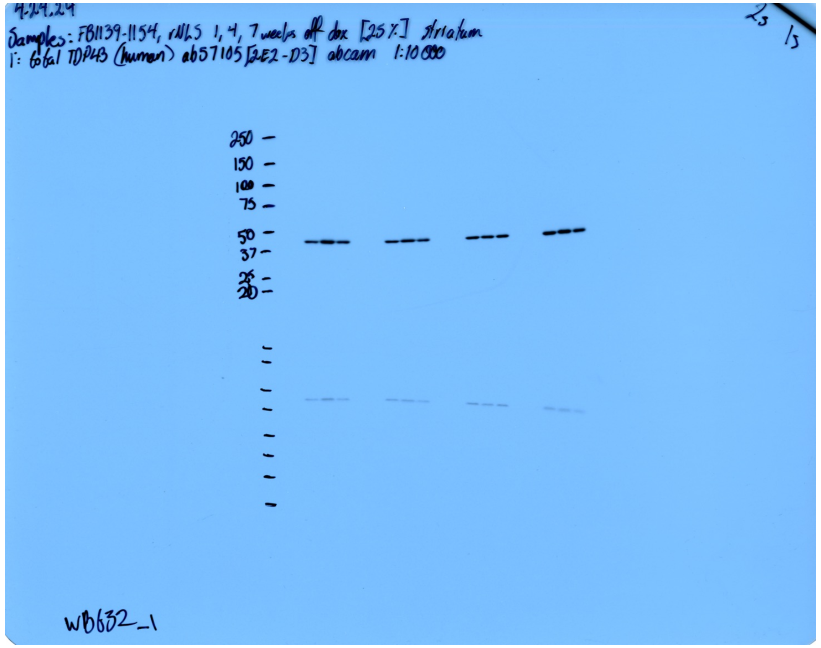


calcineurin

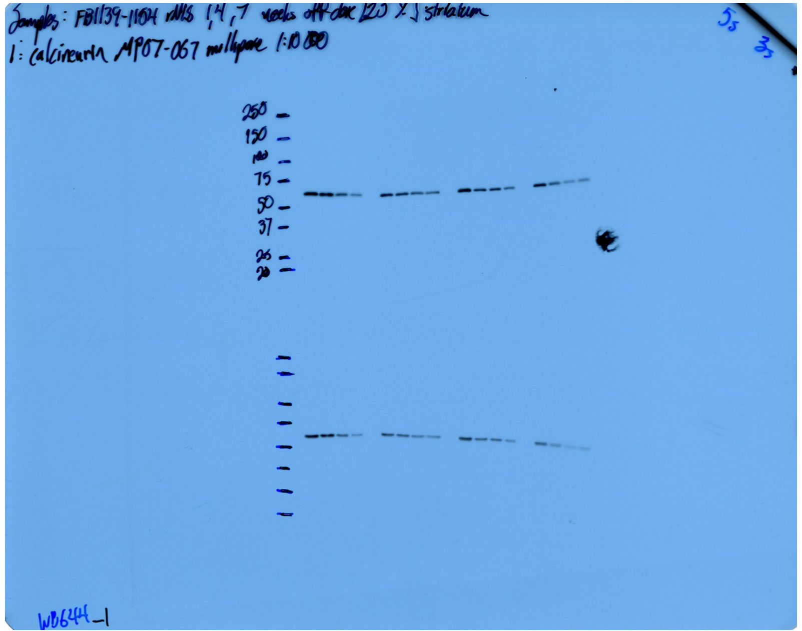


CDC7

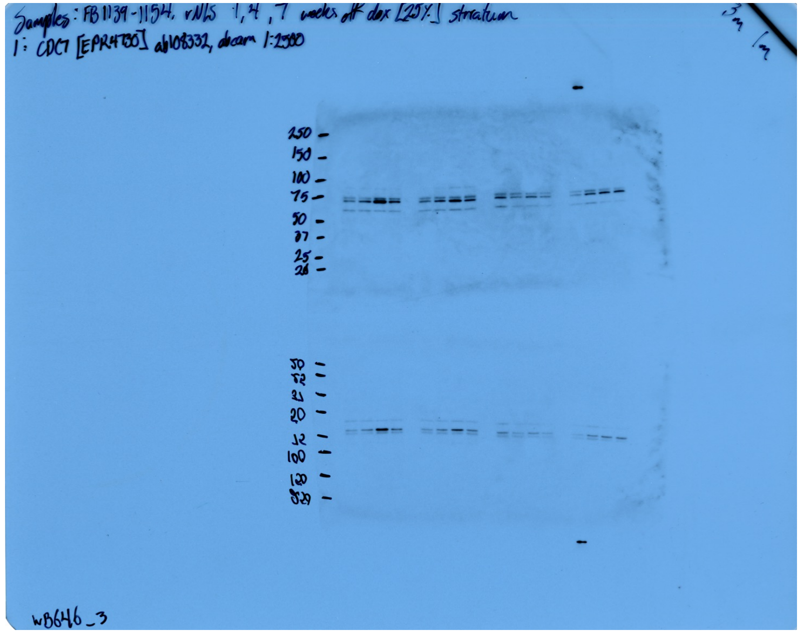


total TDP-43

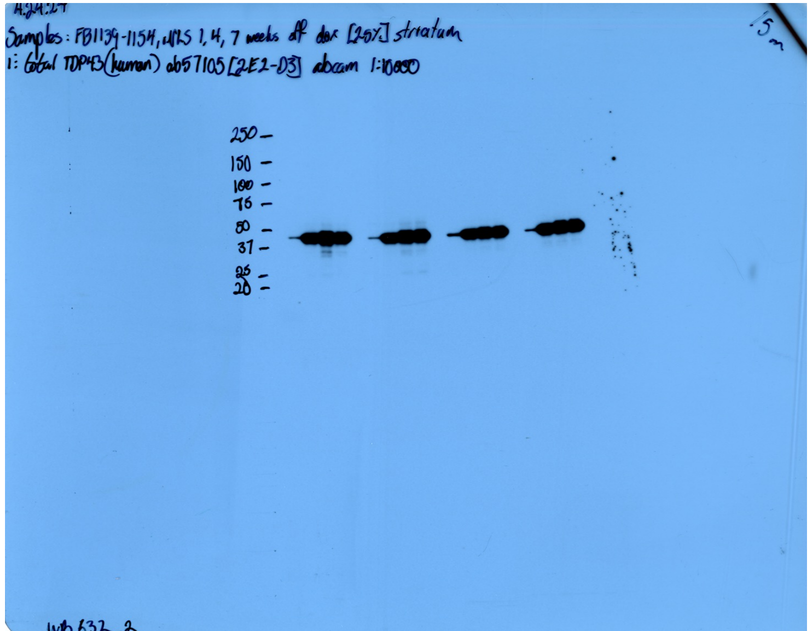

**Fig 4**

actin

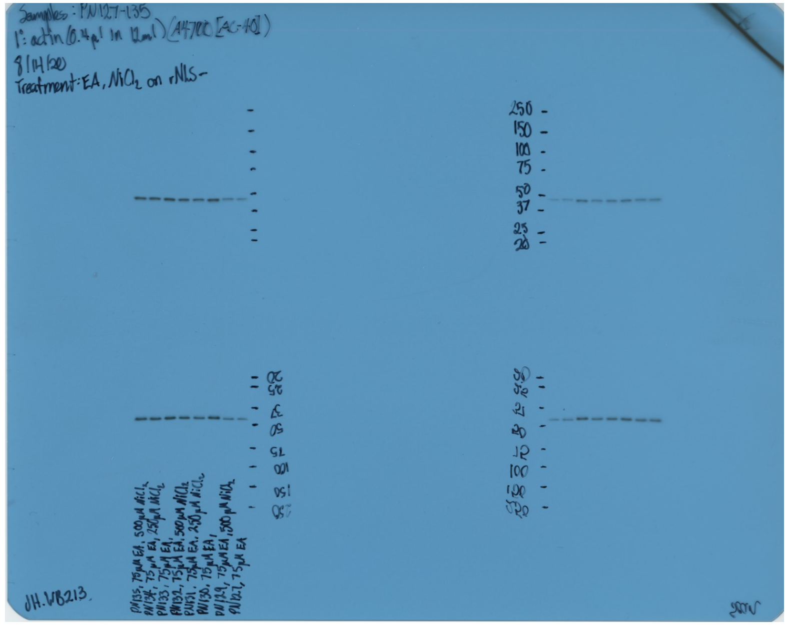

pTDP-43

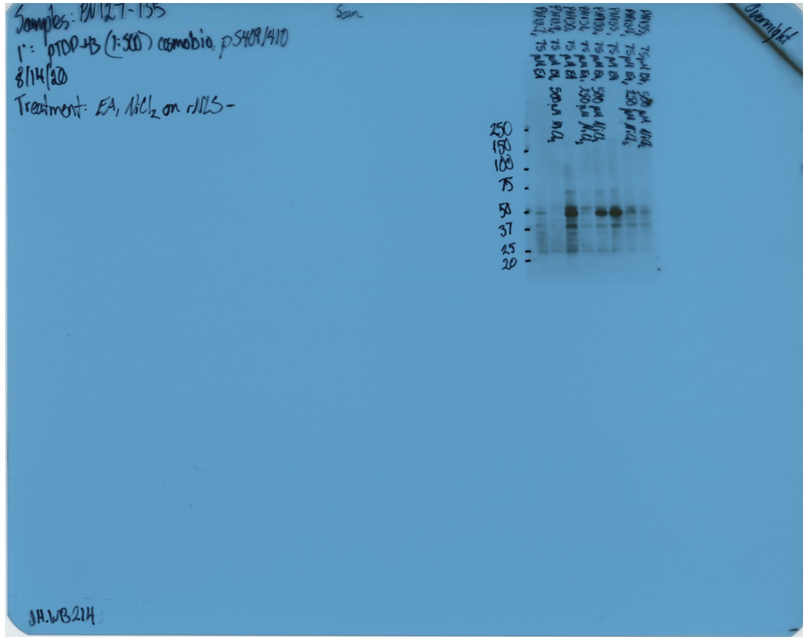

total TDP-43

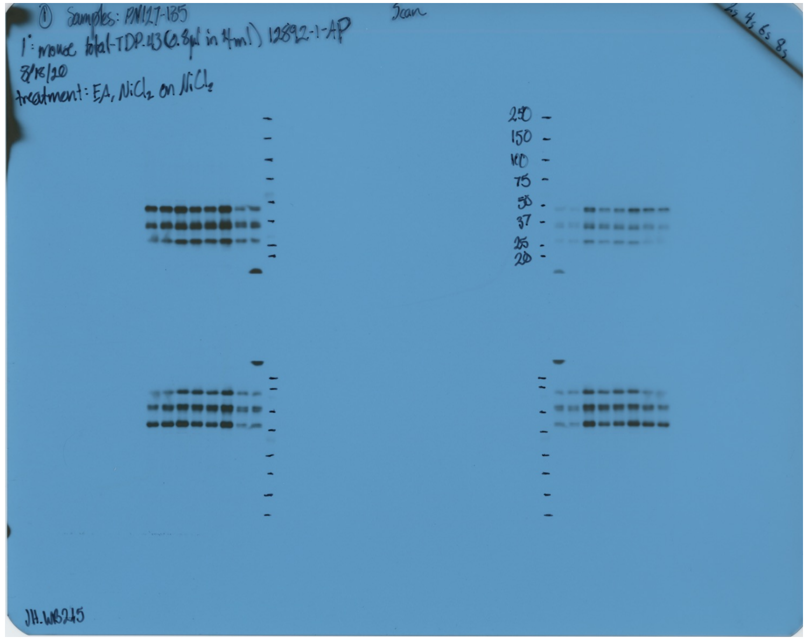

Supplement: Supplementary file 8 — Supplementary Material 8. Full immunoblots for all figures [file 40478_2025_2192_MOESM8_ESM.pdf]
